# A Web-Based Application for Personalized Ecological Momentary Assessment in Psychiatric Care: User-Centered Development of the PETRA Application

**DOI:** 10.2196/36430

**Published:** 2022-08-09

**Authors:** Fionneke M Bos, Lino von Klipstein, Ando C Emerencia, Erwin Veermans, Tom Verhage, Evelien Snippe, Bennard Doornbos, Grietje Hadders-Prins, Marieke Wichers, Harriëtte Riese

**Affiliations:** 1 Rob Giel Research Center Department of Psychiatry University Medical Center Groningen, University of Groningen Groningen Netherlands; 2 Interdisciplinary Center Psychopathology and Emotion Regulation Department of Psychiatry University Medical Center Groningen, University of Groningen Groningen Netherlands; 3 Research Support Faculty of Behavioral and Social Sciences University of Groningen Groningen Netherlands; 4 Lentis Research Groningen Netherlands

**Keywords:** eHealth, clinical implementation, ecological momentary assessment, experience sampling method, smartphone, mobile health, mHealth, personalized diaries, personalized psychiatry, client-tailored, cocreation, shared decision-making, mobile phone

## Abstract

**Background:**

Smartphone self-monitoring of mood, symptoms, and contextual factors through ecological momentary assessment (EMA) provides insights into the daily lives of people undergoing psychiatric treatment. Therefore, EMA has the potential to improve their care. To integrate EMA into treatment, a clinical tool that helps clients and clinicians create personalized EMA diaries and interpret the gathered data is needed.

**Objective:**

This study aimed to develop a web-based application for personalized EMA in specialized psychiatric care in close collaboration with all stakeholders (ie, clients, clinicians, researchers, and software developers).

**Methods:**

The participants were 52 clients with mood, anxiety, and psychotic disorders and 45 clinicians (psychiatrists, psychologists, and psychiatric nurses). We engaged them in interviews, focus groups, and usability sessions to determine the requirements for an EMA web application and repeatedly obtained feedback on iteratively improved high-fidelity EMA web application prototypes. We used human-centered design principles to determine important requirements for the web application and designed high-fidelity prototypes that were continuously re-evaluated and adapted.

**Results:**

The iterative development process resulted in Personalized Treatment by Real-time Assessment (PETRA), which is a scientifically grounded web application for the integration of personalized EMA in Dutch clinical care. PETRA includes a decision aid to support clients and clinicians with constructing personalized EMA diaries, an EMA diary item repository, an SMS text message–based diary delivery system, and a feedback module for visualizing the gathered EMA data. PETRA is integrated into electronic health record systems to ensure ease of use and sustainable integration in clinical care and adheres to privacy regulations.

**Conclusions:**

PETRA was built to fulfill the needs of clients and clinicians for a user-friendly and personalized EMA tool embedded in routine psychiatric care. PETRA is unique in this codevelopment process, its extensive but user-friendly personalization options, its integration into electronic health record systems, its transdiagnostic focus, and its strong scientific foundation in the design of EMA diaries and feedback. The clinical effectiveness of integrating personalized diaries via PETRA into care requires further research. As such, PETRA paves the way for a systematic investigation of the utility of personalized EMA for routine mental health care.

## Introduction

### Background

Ecological momentary assessment (EMA; also referred to as the experience sampling method or ambulatory monitoring) [1], is an increasingly popular self-monitoring method to gain insight into daily life experiences relevant to mental health problems. With EMA, clients monitor their daily life *as it is lived* via short questionnaires (ie, diaries) on their smartphones. Several times per day, they rate their experienced symptoms, affect, thoughts, activities, and (social) contexts [2]. In research, EMA has already been applied to improve our understanding of mental disorders by examining how symptoms fluctuate in daily life, in relation to stressful and rewarding events, and in a diverse range of mental disorders [3-6]. Increasingly, researchers and scientists are recognizing the potential of EMA as a tool for improving mental health care [7-10]. Self-monitoring through EMA and visualizing the data in EMA feedback may offer clients and clinicians relevant insights into the person-specific mechanisms that contribute to clients’ mental health problems [8].

### EMA as a Self-monitoring Tool

In many medical disciplines, self-monitoring of, for example, daily blood pressure or glucose levels, is already a common practice and is informative for diagnosis and treatment planning [11,12]. Similarly, mental health treatment may benefit from the self-monitoring of daily symptoms, (social) contextual experiences, and treatment-related factors [13]. Indeed, for this reason, many mental health treatments such as cognitive behavioral therapy already use some form of (paper-and-pencil) self-monitoring [14], and routine outcome monitoring is well established [15]. However, EMA is both more detailed and more frequent than existing monitoring methods. Typically, EMA assesses momentary experiences (“right now, I feel cheerful”), which can be rated on a visual analog scale ranging from 0 to 100 (“not at all” to “very much,” respectively). In mental health care, momentary experiences can pertain to symptoms (eg, sadness, restlessness, or hearing voices) or other factors relevant to treatment (eg, harmful or helpful thoughts and behaviors). The number of EMA assessments per day, as well as the timing and duration of the assessments, depend on the goal of the EMA diary and the experienced burden of completing the diaries. Most EMA studies thus far have used 3 to 10 assessments per day for 1 to 2 weeks [16,17], whereas some have monitored for up to 4 months [18].

### Clinical Promise of EMA

Self-monitoring through EMA is suggested to improve clients’ self-management by increasing their insights into their well-being. By frequently reflecting on one’s symptoms and learning what types of activities or situations positively or negatively influence well-being, clients may become more in control [19]. As such, EMA self-monitoring may be an intervention in itself. In addition, EMA data can be visualized to enhance the understanding of clients’ personal mechanisms that contribute to mental health problems. Such EMA feedback could be as simple as demonstrating variability in affect or showing contexts in which clients have complaints. As EMA is suggested to be unaffected by memory biases [20], such feedback could provide a more reliable overview of how clients fared between treatment sessions. This could improve shared decision-making and the therapeutic alliance (ie, the client-clinician relationship, involving an agreement on the goals and tasks of treatment, as well as their interpersonal bonds [21]) [10,19]. Therefore, EMA feedback could form the basis of a more collaborative approach to diagnosis and intervention, where clients and therapists use EMA data to decide together on the next treatment step.

### Empirical Evidence for Clinical Effectiveness of EMA

The first empirical investigations into the clinical effectiveness of EMA are promising. In qualitative research, clients and clinicians describe the beneficial effects of EMA on client self-management, therapeutic alliance, and treatment effectiveness [22-25]. This is supported by a clinical trial demonstrating that adding EMA monitoring and feedback to antidepressant treatment was more effective in reducing depressive symptoms than using antidepressant medication alone [26], and it improved clients’ feelings of empowerment [27], emotion differentiation [28], and behavioral change [29]. However, 2 other clinical trials did not replicate these beneficial effects in individuals with depression in routine care [30] and individuals reporting a loss of interest and pleasure (anhedonia) [31]. This discrepancy may be because in clinical trials thus far, EMA diaries have been standardized for all clients. This insufficiently relates EMA feedback to clients’ treatment goals, making the EMA less effective, and may increase the burden for clients, who have to frequently answer questions that are irrelevant to their current situation [32]. Therefore, personalization of the EMA seems to be a core requirement for successful clinical implementation.

### Developing Web-Based EMA Technology

Given that clients, clinicians, and researchers align in their positive evaluation of the clinical utility of EMA, the field needs a flexible, user-friendly, and evidence-based application for the use of personalized EMA diaries in mental health care. More specifically, this entails the development of a digital infrastructure that enables clients and clinicians to intuitively construct personalized EMA diaries, deliver them to clients’ smartphones, and dynamically visualize the gathered EMA data.

In this paper, we describe the development of Personalized Treatment by Real-time Assessment (PETRA), a web-based application that enables the use of personalized EMA diaries in Dutch mental health care. PETRA was built into electronic health record (EHR) systems to ensure sustainability and easy access. The web application comprises a decision aid to help clients and clinicians construct personalized EMA diaries, an EMA diary item repository, a diary delivery system, and a feedback module to visualize the gathered EMA data. PETRA was developed in close collaboration with clients and clinicians as they will be the primary users. Involving them early in the development process is crucial to ensure the uptake of PETRA.

## Methods

### Study Design

PETRA was developed using the road map of the Center for eHealth Research (CeHRes) of the University of Twente, the Netherlands [33]. The road map comprises 5 iterative phases. In the first 2 phases of the CeHRes road map (contextual inquiry and value specification), we conducted focus groups and interviews to identify the needs and requirements of the relevant stakeholders, clients and clinicians, for a personalized diary web application [34]. The next phase (design) comprised an iterative design process in which prototypes were developed, evaluated, and adapted frequently based on feedback from clients and clinicians [35]. In this paper, we describe the results of these 3 phases and the resulting PETRA web application. The final 2 phases of the CeHRes road map (operationalization and summative evaluation) will involve implementing the web application and evaluating its use in practice.

**Figure 1:**
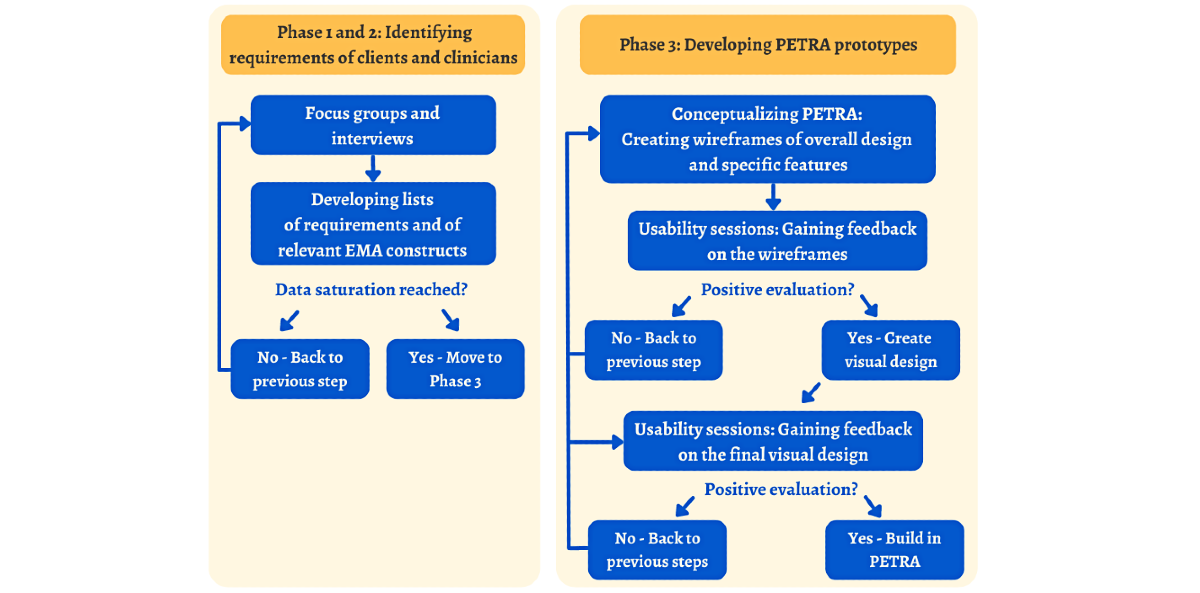
Overview of the developmental phases of PETRA (Personalized Treatment by Real-time Assessment). EMA: ecological momentary assessment.

During each of these phases, stakeholders were actively involved to ensure their perspectives are integrated into PETRA. The CeHRes road map follows human-centered design principles, attempting to understand the needs of users and actively involving them in designing solutions to meet these needs [36]. Therefore, the PETRA project team was multidisciplinary and comprised the core scientific team; a scientific programmer; the RoQua development team to ensure sustainable embedding in existing mental health care software; a user experience (UX) designer to integrate the needs of all stakeholders; and client and clinician representatives engaged in interviews, focus groups, and usability sessions. All team members critically reviewed the prototypes, which were then continuously adapted and re-evaluated (Figure 1).

### Setting and Participants

The PETRA web application was developed from 2016 to 2022 at the Department of Psychiatry, University Medical Center Groningen (the Netherlands), facilitated by iLab, a collaborative initiative to implement scientific innovations into clinical practice [37]. PETRA’s software was built into RoQua [38], a nonprofit web-based questionnaire system for research and care. RoQua is fully integrated into the EHR systems of several mental health care facilities in the Netherlands, which are part of the Rob Giel Research Center (RGOc) [39]. The facilities participating in this collective mostly provide secondary or tertiary (specialist) mental health care services to clients presenting with a diverse range of mental disorders, such as major depression, bipolar disorder, anxiety disorders, and psychosis.

### Participant Recruitment

Participants comprised a convenience sample of clients and clinicians recruited from RGOc facilities, who took part in interviews, focus groups, and usability sessions. All clients with a (self-reported) history of mental health problems (regardless of type) were eligible to participate in the study. Similarly, all clinicians working in an RGOc facility were eligible. Clinicians could be psychiatrists, psychologists, or psychiatric nurses. Participant characteristics for the first 2 phases can be found in the studies by Bos et al [22,23] but were not systematically collected in the design phase. The clients were informed of the PETRA project by the RGOc coordinator of the client representatives or their clinician. If interested, clients received a flyer and, after providing consent, were invited for an interview, focus group, or usability session. The clients were reimbursed for their time (€10 [US $10.18] per hour). Clinicians were invited based on prior participation in ongoing diary studies or their expressed interest in PETRA during (department) presentations. Both clients and clinicians were invited to join multiple sessions to offer feedback on the adaptations that were made.

### Ethics Approval

The University Medical Center Groningen Medical Ethics Committee exempted this research from a full review (reference number 201900401). Clients signed a written informed consent form to participate in the focus groups, interviews, and usability sessions.

### Phases 1 and 2: Identifying the Requirements of Clients and Clinicians

Phases 1 and 2 aimed to identify perceived problems in the status quo of mental health treatment, perceived advantages and challenges of personalized electronic diaries, and core requirements for the EMA web application to be developed. In-depth qualitative interviews and focus groups were conducted with 40 clients and 27 clinicians between June 2016 and March 2018 on the perceived utility of EMA for psychiatric care and perceived important requirements for a clinical EMA tool. Focus groups with clients or clinicians were conducted in groups of 3 to 7 participants until data saturation was reached (ie, no new themes emerged). The focus groups and interviews were part of 2 qualitative studies that broadly focused on identifying applications of EMA in diverse clinical contexts and implementation requirements. For more details, we refer the reader to previous studies [22,23] (for interview guides, see Table S1 in Multimedia Appendix 1). In this paper, we specifically discuss the resulting recommendations for the development of an EMA tool on which PETRA was based. Furthermore, in this phase, we compiled a list of potentially relevant constructs to assess personalized EMA diaries. On the basis of previous EMA research and subsequent suggestions by clients and clinicians, each construct was linked to related EMA diary items (eg, “I feel anxious” for the construct “Anxiety”).

### Phase 3: Development of PETRA Prototypes

#### Overview

On the basis of the recommendations in phases 1 and 2, we designed prototypes for the web-based personalized EMA tool PETRA. These were continuously tested and redesigned in usability sessions with clients and clinicians held from May 2019 to August 2021. Owing to the COVID-19 pandemic, all usability sessions after March 2020 were conducted via video calls (for interview guides, see Table S1 in Multimedia Appendix 1). At each session, the researcher FB and UX designer TV were present. After each session, they wrote a report with the main recommendations. The participants were invited to provide feedback on this report.

#### Sessions With Clinicians

On the basis of phases 1 and 2, clinicians were considered the primary users of the decision aid and feedback module: they would need to integrate PETRA into their working routines and introduce it to clients. Therefore, most of the usability sessions were held with clinicians. A total of 18 clinicians participated in ≥1 of the 31 usability sessions (5/18, 28% were men). Approximately 77% (24/31) of the sessions were held individually, and 23% (7/31) of the sessions took place in groups of 2 to 3 clinicians. Most clinicians were psychologists (10/18, 56%), followed by psychiatrists (4/18, 22%) and psychiatric nurses (4/18, 22%). Of the 18 clinicians, 7 (39%) participated once, 8 (44%) participated twice, and 3 (17%) participated thrice. During each session, clinicians were asked to describe their thoughts and expectations regarding EMA and their requirements for a diary web application. Subsequently, the clinicians were walked through a digital prototype of the PETRA web application. They were encouraged to describe what they saw on the screen and provide their initial thoughts on each page (the think-aloud method). When the clinicians offered suggestions for improvement, follow-up questions were asked until an in-depth understanding of the proposed adaptation was reached. The clinicians were also invited to explain how they would use PETRA in treatment and to suggest new features or feedback types.

#### Sessions With Clients

A total of 12 clients participated in ≥1 of the 4 usability sessions, which were similar to those with the clinicians. Clients were currently or had been previously in treatment for mood, psychotic, or anxiety disorders (8/12, 67% were men). Of the 12 clients, 9 (75%) participated in 1 usability session, and 3 (25%) participated in 2 sessions. The sessions took place in groups of 3 to 7 clients. One of the sessions specifically focused on designing low-fidelity (paper-and-pencil) prototypes for EMA diary feedback. The other usability sessions were used to obtain feedback on the digital high-fidelity prototypes of the PETRA web application. Feedback was sought on the workflow and its intended use in treatment.

### Data Analysis

The interviews and focus groups were transcribed and analyzed thematically using the Qualitative Analysis Guide of Leuven [40]. Briefly, this approach involves the identification of important themes within qualitative data, which are then iteratively verified against the transcripts. Further details are provided elsewhere [22,23]. The resulting themes regarding the requirements of a clinical EMA tool are described in the Results section.

**Figure 2:**
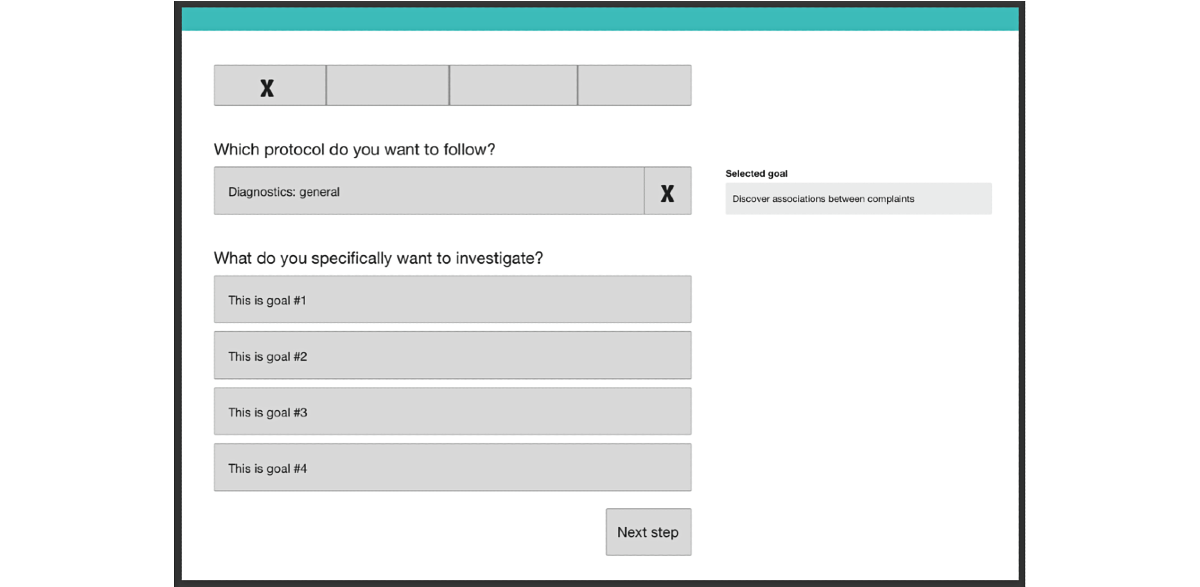
The first wireframe that was designed of the first page of the decision aid, on which participants select the goal of the EMA diary. The usability sessions indicated a need for an introduction and a more detailed progress menu. More detailed wireframes can be found here [41]. EMA: ecological momentary assessment.

**Figure 3:**
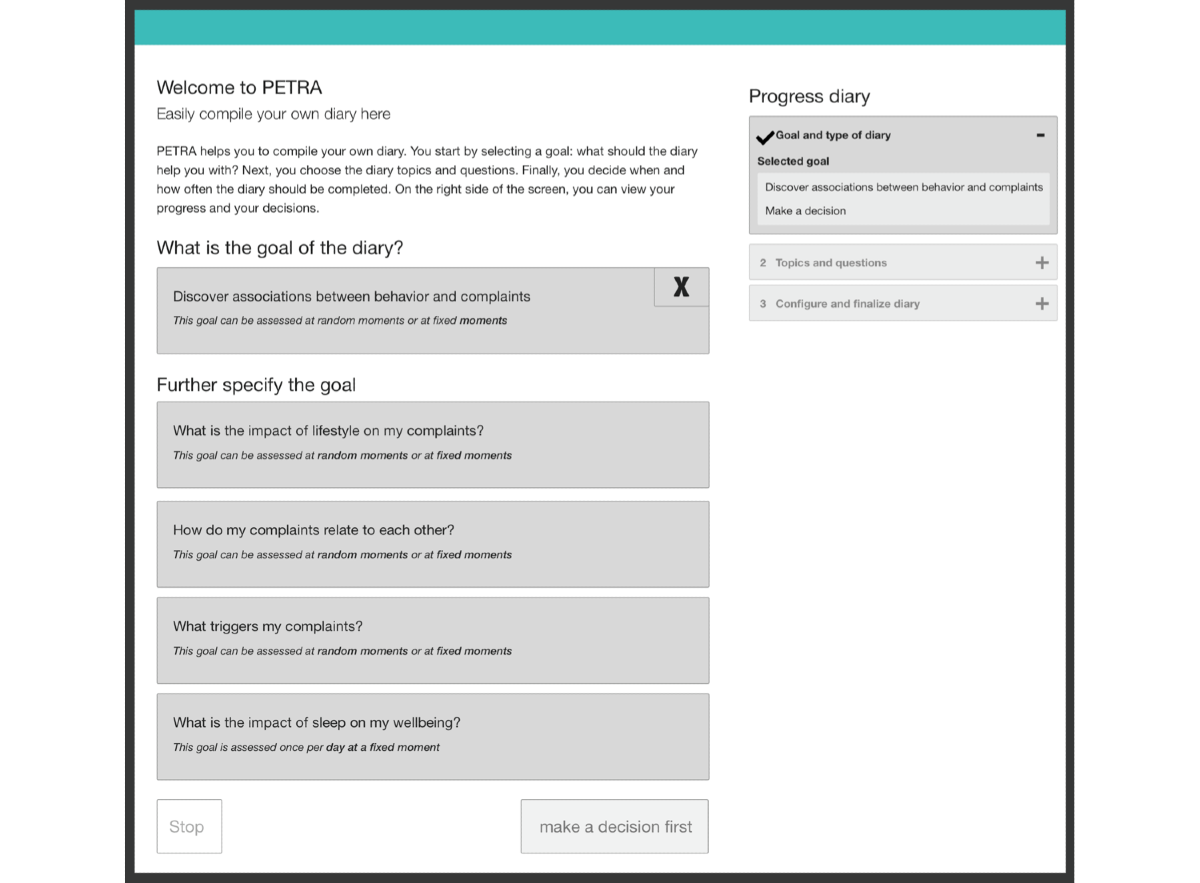
The ninth wireframe that was designed of the first page of the decision aid, on which participants select the goal of the EMA diary. As suggested in usability sessions, it incorporates an introduction and a more detailed progress menu. More detailed wireframes can be found here [41]. EMA: ecological momentary assessment.

**Figure 4:**
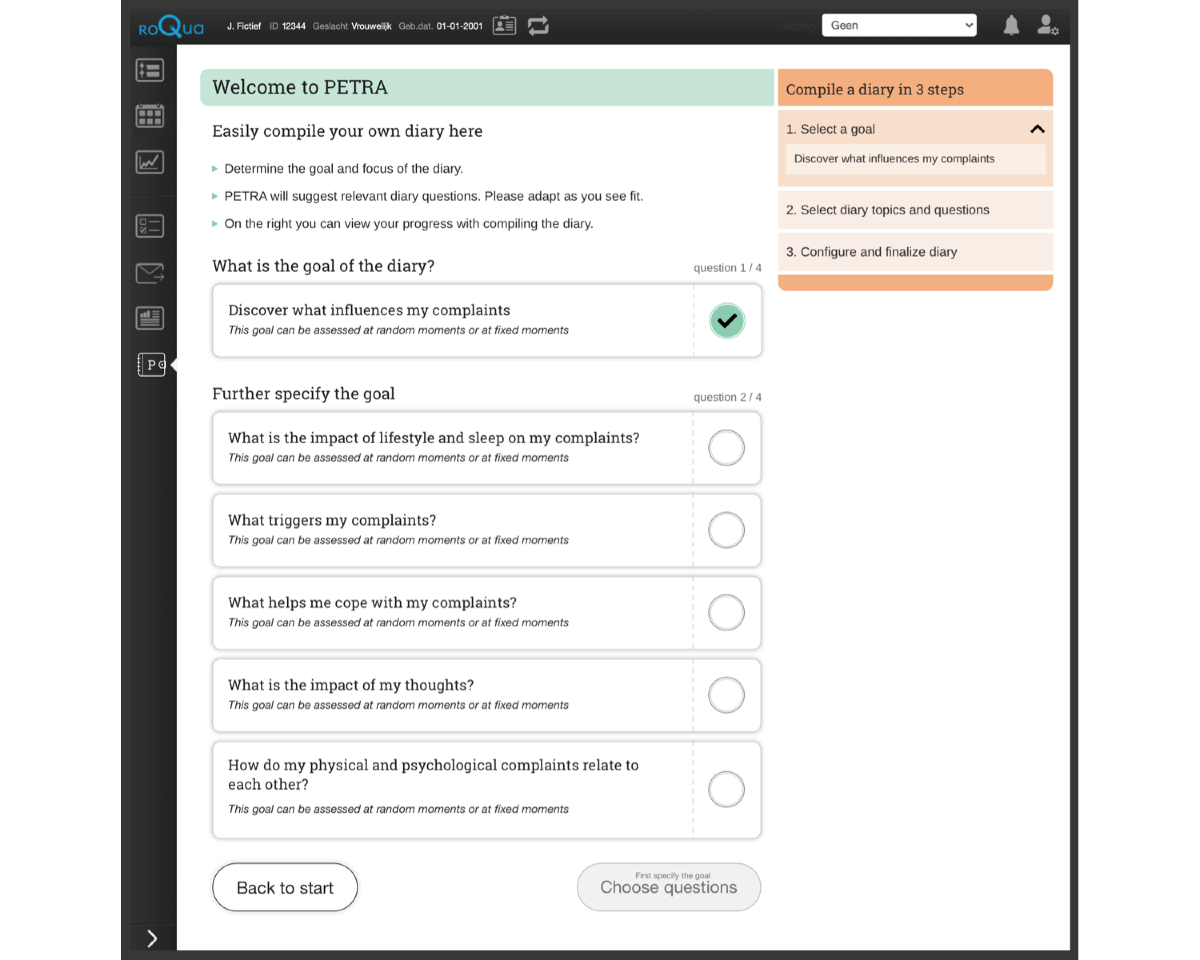
The final (visual) design of the first page of the decision aid, on which participants select the goal of the EMA diary. More detailed wireframes can be found here [41]. EMA: ecological momentary assessment.

After each usability session, wireframes were created with the proposed changes to the PETRA web application (see Figures 2-4 for example wireframes or a slide-by-slide overview on the PETRA website [41]). A wireframe is a blueprint that explicates the flow throughout the web application. In total, 9 low-fidelity wireframes of the overarching structure and workflow of PETRA were designed, each version improving upon the previous one. The final ninth wireframe was used for the next stage, which focused on determining the types of content, visuals, and graphs depicted on the screens. This stage comprised 15 high-fidelity wireframes, which were again improved iteratively.

## Results

### Phases 1 and 2: Core Requirements of PETRA

#### Overview

The first 2 phases of the CeHRes road map resulted in an in-depth overview of clients’ and clinicians’ perceptions of perceived problems in the status quo of mental health treatment, perceived advantages and challenges of personalized electronic diaries, and core requirements for the EMA web application to be developed (Table 1). The core requirements were (1) extensive possibilities for personalization, (2) resource efficiency, (3) scientifically grounded and provided by a trusted source, and (4) meeting the demands regarding privacy and safe data storage. Relevant quotes can be found in Table S2 in Multimedia Appendix 1.

**Table 1:** Results of phases 1 and 2 of the Center for eHealth Research road map in the development of the PETRA (Personalized Treatment by Real-time Assessment) application.

Theme		Description
**Problems clients and clinicians identified in the status quo of mental health treatment**		
	Reliability or efficiency of current monitoring instruments	Often paper-and-pencil based and deemed less reliable and efficient Often focus too much on symptoms and fail to take important contextual factors and strengths into account Usually only administered 1 to 2 times per month; missing relevant information about clients’ daily lives throughout the day Usually not person-specific enough to be directly relevant to clients
	Insights	Limited insight into overall progress and treatment outcomes Limited insight into the effects of treatment or lifestyle adaptations Limited insight into the frequency and severity of symptoms and when they occur Limited insight into triggers and relapse signals Recall of well-being in between sessions is biased by current mood or otherwise difficult to recall for clients (recall bias)
	Therapeutic alliance	Knowledge imbalance between client and clinician, weakening the therapeutic alliance Current monitoring instruments are often not discussed in treatment, weakening the therapeutic alliance Limited contact between client and clinician in between sessions Client does not believe in the current treatment approach as the effects are not clearly visible
**Perceived advantages of personalized electronic diaries**		
	Reliability or efficiency	More reliable and efficient as assessments take place multiple times per day via smartphone and are less easily forgotten or ignored
	Insight	Offers insights into progress, treatment effects, and the flow of symptoms throughout daily life, thereby increasing client self-management Offers insights into contextual factors and personal strengths
	Therapeutic alliance	Client and clinicians share the same information and can, therefore, collaborate more easily Relevant diary feedback can more easily be integrated in treatment, strengthening the therapeutic alliance and clients’ trust in their clinician
**Perceived challenges in constructing personalized electronic diaries**		
	Diary construction	What kind of clinical questions can be answered with electronic diaries? How to formulate or select relevant and valid diary items? How to determine the number of assessments per day, balancing client burden and the number needed to answer a clinical question? How to determine the necessary diary schedule (eg, time-contingent or event-contingent) for a clinical question? How to make sure the diary maximizes its advantages and minimizes disadvantages?
	Diary feedback	How to automatically analyze and visualize the diary data without the need for statistical knowledge of the clinician? How to interpret the diary feedback in a clinical context?

#### Personalization

All clients and clinicians stressed the necessity for extensive personalization of the diary content, schedule, and duration. They suggested that clients and clinicians should be able to compile different EMA items based on the clients’ current care needs in a flexible manner. This means that EMA diaries should be transdiagnostic and adaptable in diverse stages of treatment. The alternative, where diaries are constructed based on client diagnosis, was viewed as limiting, more burdensome, and not in line with the real-world situation where many clients present with symptoms of multiple diagnoses. Furthermore, EMA diaries should not only focus on symptoms but also on personal strengths and (social) contextual factors. This need for personalization has also been highlighted in research on monitoring in general [32,42,43].

#### Resource Efficiency

Both clients and clinicians emphasized that the use of EMA in treatment should be easy, time efficient, and fit with the existing workflow of clinicians. This means that constructing personalized EMA diaries and interpreting the resulting feedback should be intuitive and cost clients and clinicians limited time, effort, and resources. This corresponds with research demonstrating that eHealth is most useful if it matches client and clinician expectations [44]. Thus, the web application should be largely automated and integrated into existing digital infrastructures used in mental health care, preferably the clinician’s EHR system.

#### Scientific Background

Clients and clinicians indicated that they found it difficult to assess the effectiveness of eHealth applications, which corresponds to eHealth research [45,46]. Therefore, they stressed the need for a web application that is scientifically grounded so that the resulting diary and feedback are valid, trustworthy, and according to scientific standards for EMA [16]. Furthermore, as clinicians are not primarily trained in the innovative methods and statistics necessary for constructing and analyzing diary data, they indicated that the web application should help them with constructing diaries and interpreting feedback.

#### Privacy

The final core requirement clients and clinicians mentioned was that the gathered diary data should be safely stored, protecting the privacy of clients, according to data protection laws (eg, the General Data Protection Regulation [GDPR] law of the European Union). Given the far-reaching personalization of the diaries, the data should be treated accordingly.

### Phase 3: Design of PETRA

#### Overview

The core requirements for a web application for personalized EMA diaries were translated into the current design of PETRA. PETRA was developed as a web-based application, built into EHR systems via RoQua. As per the suggestion of clinicians, this means that clients and clinicians do not need additional log-in data, and clients do not need to install an app on their phones, allowing easy access for both. PETRA was developed for adult clients diagnosed with mood, anxiety, or psychotic disorders receiving treatment in specialized mental health care. Proficiency in the Dutch language and client possession of a smartphone with internet access are required to use PETRA.

**Figure 5:**
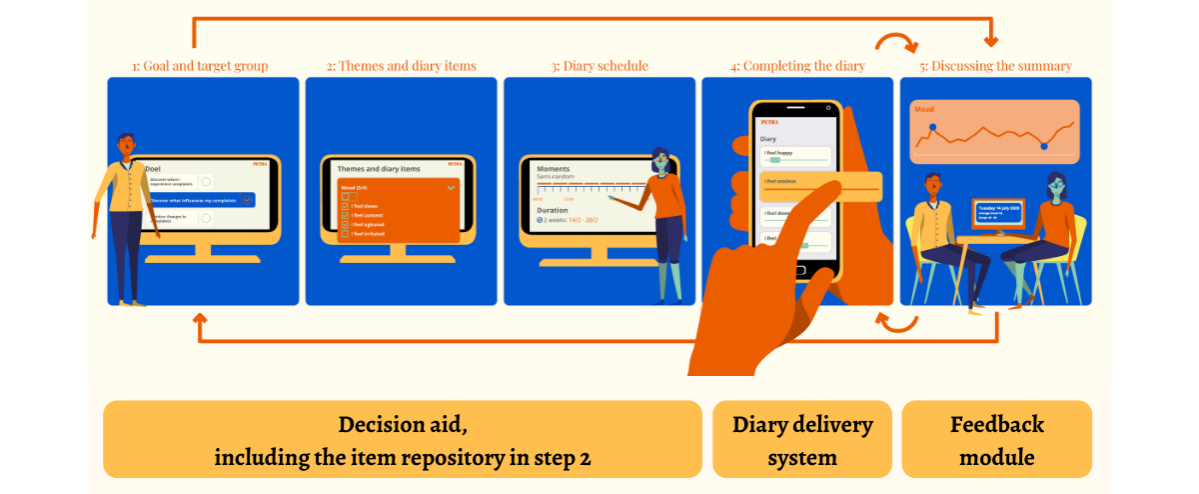
Overview of PETRA.

PETRA comprises four main parts: (1) a decision aid, (2) an item repository of diary items, (3) an SMS text message–based diary delivery system, and (4) a feedback module. An overview of the various parts of PETRA is shown in Figure 5. Each part was iteratively evaluated and adapted based on the feedback of clients and clinicians, who mostly agreed on the design of PETRA’s features. PETRA was designed to be used collaboratively: clients and clinicians together decide on the goal, content, and schedule of the EMA diary and interpret the feedback together. Clients have access to the decision aid and feedback modules via their clinician. PETRA’s steps are outlined in more detail in the following sections.

#### Decision Aid

A decision aid was developed to assist clients and clinicians in constructing personalized EMA diaries. This decision aid is intended to be used collaboratively by clients and clinicians. The following specifications can be personalized via the decision aid: (1) the content (or diary items), (2) the number of assessments per day, (3) the diary schedule (timing of the assessments), and (4) the length of the EMA monitoring period. These specifications depend on the intended goal of the diary, anticipated burden for the client, and the client’s main symptom profile.

PETRA’s decision aid was developed based on the results of phases 1 and 2 and guidelines for designing EMA studies [16,47]. First, we developed a list of clinical goals for which personalized EMA may be insightful. Such goals include, for example, gaining insights into the effects of lifestyle on well-being or monitoring the effects of medication. Each goal is linked to potentially relevant EMA items, a preferred number of assessments, a preferred diary schedule, and a preferred diary period (Tables S3-S5 in Multimedia Appendix 1). This matching process was based on clinical EMA research demonstrating the requirements for different types of research questions. For example, if gaining insights into contextual factors is the primary goal, the best practice in EMA research is to opt for a semirandom diary schedule, in which the assessments are randomly generated within multiple predefined time blocks. This is generally considered the most ecologically valid way of gaining an overview of someone’s activities and events during the day [48].

In addition to the goal, clients and clinicians select the symptom profile they wish to focus on. We developed a list of prevalent symptom profiles in specialist mental health care (eg, depression, anxiety, and psychosis). Each symptom profile was linked to potentially relevant EMA items. For example, relevant constructs for clients in depression treatment could be mood, anhedonia, worry, self-esteem, and resilience.

The decision aid first prompts clients and clinicians to decide on the clinical goal, symptom profile, and preferred diary schedule (see Figure 5 or the PETRA website for a video [49]). In the next 2 steps, based on these decisions, PETRA automatically preselects the relevant EMA items and proposes a diary schedule. This preselection can be adjusted according to personal preferences while remaining within valid boundaries (eg, if the goal is to assess the relationship between activities and symptoms, only a semirandom schedule of at least five assessments per day can be selected [48]). Clients and clinicians can deselect irrelevant items or select additional items. The PETRA decision aid further includes a preview function, which visualizes the diary as the patient views it on their phone, and a *burden indicator*, which is a gradient bar that changes color according to the number of items in the diary to remind clients and clinicians to discuss the burden when constructing diaries (S6 and S7 in Multimedia Appendix 1 [16,17,47,50,51]). Although research currently offers no clear-cut guidelines on the diary length and associated burden [17,50], a study demonstrated that 60 items per diary were perceived as more burdensome than 30 [51], and another study showed that EMA studies, on average, comprised 30 items [16]. It is likely that the number of items and associated burden differs between, as well as within, individuals and across populations and treatment stages. Therefore, the burden indicator was based on our experience in designing EMA studies and will be updated based on the UXs and emerging research.

#### EMA Item Repository

The EMA item repository was developed as clients and clinicians indicated a preference for a *menu* in which they could select clinically relevant constructs (eg, mood or hearing voices) that automatically tie into preworded items. Although the EMA field is yet to establish the validity of EMA items, best practices have been developed in EMA research on the wording of EMA items [16,47,50]. The PETRA items were developed based on these best practices.

The EMA item repository was developed by the scientific team based on extensive experience with the items used in EMA studies and subsequent evaluations and suggestions by clients and clinicians. First, we determined potentially relevant constructs to be assessed; that is, constructs pertaining to symptoms, strengths, and contextual factors. For each construct, we formulated 4 to 10 diary items.

This resulted in four item sets based on the EMA assessment schedule: (1) at semirandom moments (128 items), (2) at fixed moments (126 items), (3) once daily in the evening (62 items), and (4) once daily in the morning (18 items). EMA assessments can occur either at fixed time points (eg, at noon) or at random points in predefined intervals (eg, somewhere between 10:30 AM and noon), termed fixed or semirandom EMA designs, respectively. Fixed designs are believed to be less burdensome for clients, whereas semirandom designs supposedly provide a more representative overview of daily experiences [2,48]. The item sets were developed as experiences can be expected to operate on different timescales and, therefore, require different wording. For example, mood usually fluctuates throughout the day and requires multiple assessments for valid estimation. However, excessive buying, a symptom of mania, may only vary from day to day and can be sufficiently assessed only in the evening. Most items are answered on 0 to 100 visual analog scales (ranging from “not at all” to “very much”), some via selecting categorical multiple-choice checkboxes (eg, indicating activities, coping strategies, or locations), and some via entering text (eg, descriptions of events). The full lists of EMA items are available in item S9 in Multimedia Appendix 1 [16-18,22,23,26,37,42,47,50-72] (Bos, F, unpublished data, May 2022; Jenner, JGL, unpublished data, October 2006; Bringmann, L, unpublished data, January 2021). In addition to the item sets, clients and clinicians can also formulate a limited number of person-specific items.

Semirandom and fixed diary schedules are mutually exclusive. Once-daily items are often an informative addition to these more frequent assessment schedules. Therefore, in the decision aid, the constructs of the once-per-day schedules are integrated within the construct structure of the semirandom and fixed schedules. This ensures that clients and clinicians can select items based on the construct. For example, the construct *(hypo)mania* provides items assessed in the moment (semirandom or fixed), as well as morning and evening items. Although the EMA items are grouped into constructs in the decision aid, they are reordered in the actual diary to balance positive and negative items and ensure that more fleeting momentary emotions are assessed before contextual experiences [16,50].

#### EMA Delivery System

Once clients and clinicians have constructed the EMA diary, clients receive SMS text messages on their personal smartphones. The messages are sent according to the chosen diary schedules and contain a link to the EMA diary items, which are presented and filled in via the browser. Clients receive reminders if the diary has not been completed within 20 minutes, and the link is disabled after 30 minutes (semirandom and fixed schedules) or 3 hours (once-daily schedules).

#### Feedback Module

**Figure 6:**
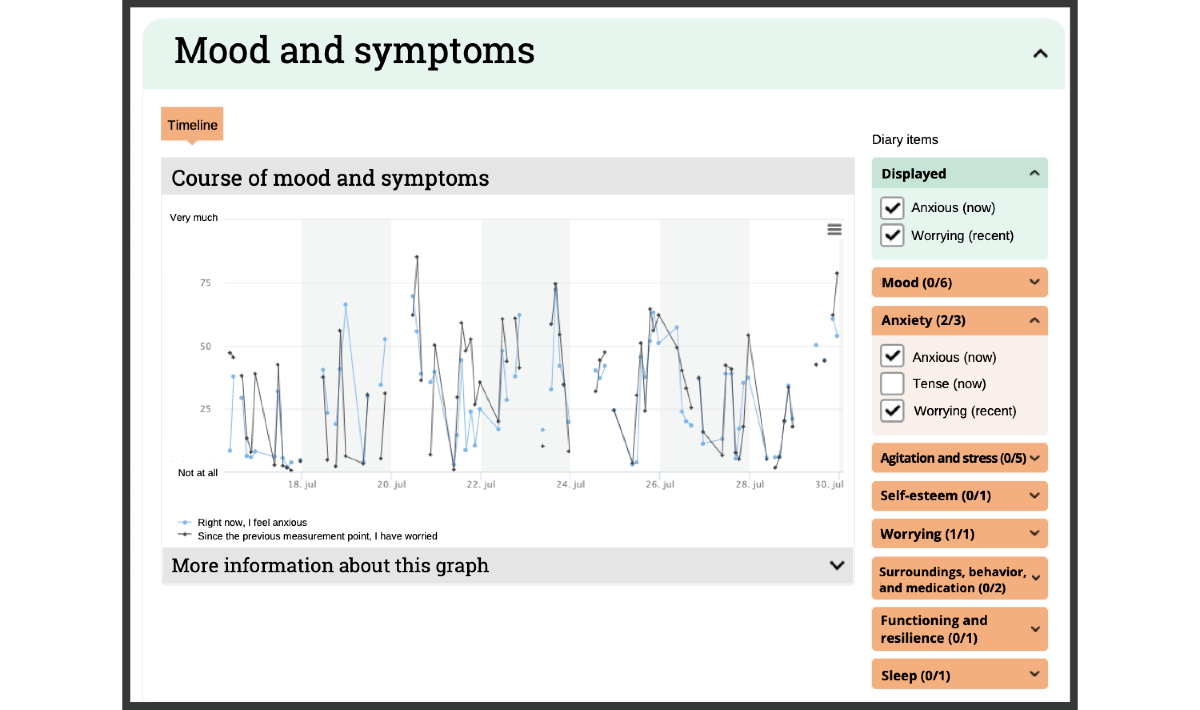
An interactive graph on the variation in mood and symptoms in the feedback module. All continuous ecological momentary assessment (EMA) diary items can be selected from the menu on the right side of the screen. Clicking on any of the assessment points will activate a sliding pane depicted in Figure 7. For a video, see here [49].

**Figure 7:**
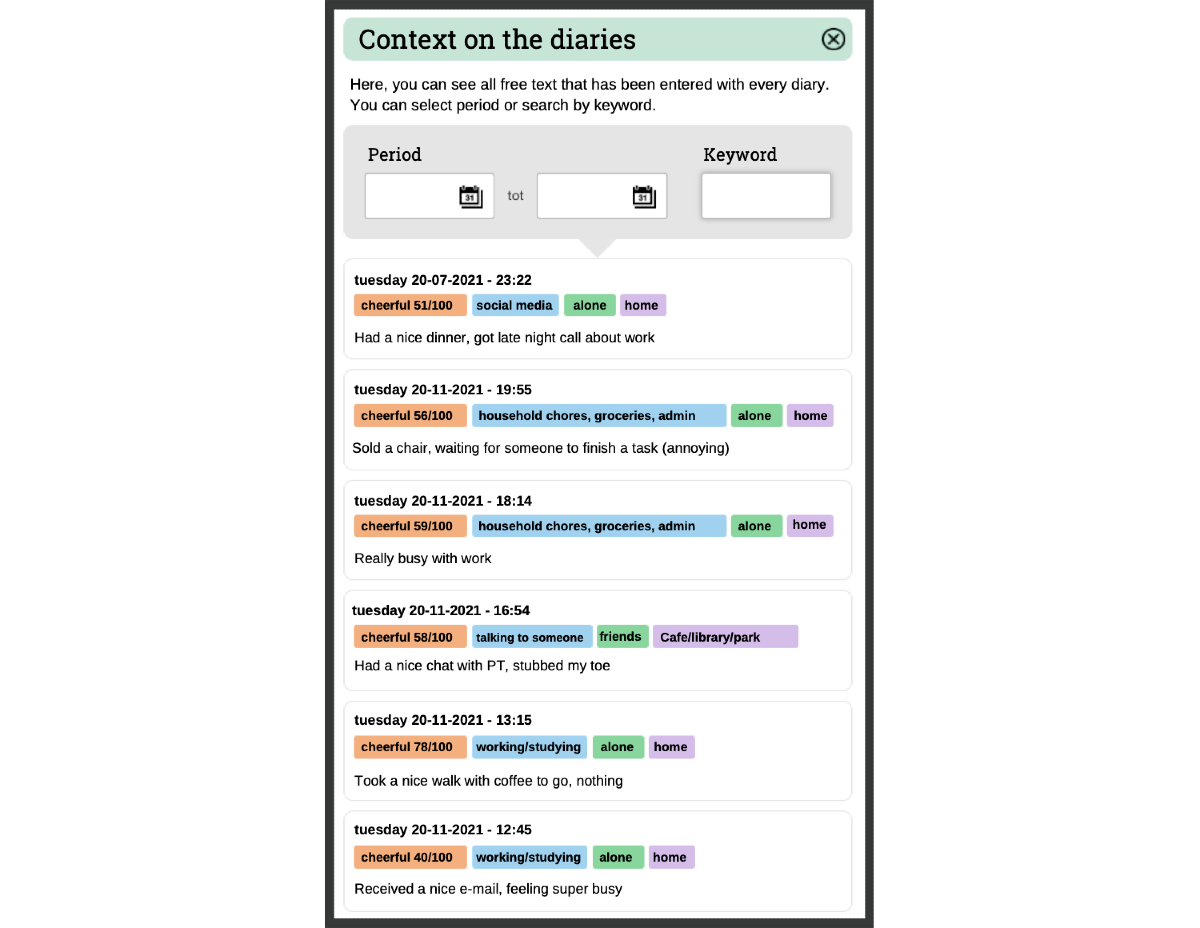
Sliding panel in the feedback module that provides relevant contextual information for a specific moment in the time series and surrounding moments. Moments are selected by clicking on assessment points in the graph depicted in Figure 6. For a video, see here [49].

**Figure 8:**
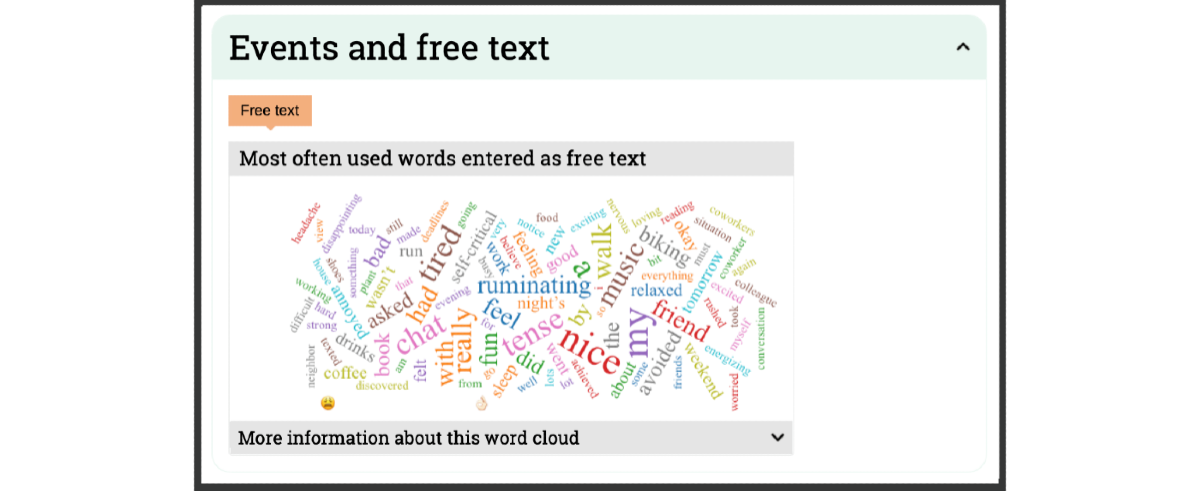
A word cloud of all freely entered text in the feedback module. Clicking on a word provides more context on when this word was used. For a video, see here [49].

The gathered EMA data for each client are visualized in the PETRA feedback module, which clients and their clinicians can discuss during a regular treatment session (see Figures 6-8 or the PETRA website for a video [49]). This module is grouped around three themes identified by clients and clinicians as relevant to psychiatric treatment: the fluctuations and changes in symptoms and strengths over time, associations between (social) contexts and symptoms and strengths, and the impact of pleasant and unpleasant events (see item S8 in Multimedia Appendix 1 for more details). The early versions of the feedback module were based on the feedback module developed in the Therap-i trial [52]. The PETRA feedback module is updated in real time and offers dynamic feedback options, and each graph can be adapted and can display any of the assessed EMA items.

Clients and clinicians indicated that the understanding of EMA data is greatly facilitated by qualitative descriptions of the context provided by clients. Therefore, clients are actively encouraged to provide such descriptions. In the feedback module, these qualitative evaluations can be linked to high and low scores to provide contextual information on moments the client experiences high or low symptoms. Furthermore, all entered text is summarized in word clouds to intuitively summarize the main themes that emerged from the qualitative data and provide context for when these themes emerged. The PETRA feedback is descriptive as statistical analysis of diary data was considered too complex, unclear, and vulnerable to misinterpretation [73,74]. However, the modular structure of the feedback module ensures that novel feedback can be easily added in later stages. For example, if empirical evidence shows that certain patterns in EMA data can be linked to treatment response or outcome, PETRA can offer this information directly in its feedback module [75-77]. Finally, the module has a report function, which enables users to save graphs and comments (eg, therapy notes) for later use.

## Discussion

### Principal Findings

In this paper, we outline the development of PETRA, a web-based application for personalized EMA in Dutch psychiatric care. Interviews, focus groups, and usability sessions with clients and clinicians demonstrated that they expected an added value of integrating personalized EMA into treatment. Crucial requirements entailed a personalized and user-friendly diary tool built on scientific foundations, which adheres to privacy regulations. The subsequent thorough cocreation design process ensured that PETRA was considered intuitive, user-friendly, and useful for clients and clinicians.

By involving clients and clinicians from the start of the development, PETRA can be considered a significant advancement of current EMA (web-based) applications that are mostly targeted at researchers. By systematically integrating the perspectives of clients and clinicians with those of researchers and software developers, the PETRA web application has promise as a tool for assisting both clients and clinicians in the personalized treatment of mental health problems.

### Limitations

In this project, we attempted to include a diverse group of clients and clinicians with differing levels of interest in using personalized EMA for treatment. However, this was a relatively small sample, and participants were selected based on their interest in EMA. Therefore, it cannot be ruled out that we overestimated the eventual uptake in clinical practice. As EMA requires significant time and resources from both clients and clinicians, it may not appeal to everyone [78,79], which was also highlighted in the focus groups and interviews. However, research indicates that EMA is feasible for diverse psychiatric populations [47,80,81], including individuals diagnosed with depression [26,27,82], bipolar disorder [83,84], anxiety [85], and psychotic [53,86,87] disorders, from youth [88,89] to older adult populations [90]. Feasibility will likely not depend on diagnosis but rather on the intrinsic motivation of clients, symptom severity, and stage of care [22,23].

Furthermore, there are some limitations that pertain specifically to the PETRA tool. First, PETRA is only accessible to clients via their clinicians via their EHRs. This can be a psychiatrist, psychologist, or psychiatric nurse affiliated with a RoQua mental health care facility. We are currently developing client access to PETRA directly via their personal health record system to enable clients to interpret feedback independently of their clinician. This is in line with clients’ desire to have access to their own data [23]. Second, proficiency in the Dutch language and client possession of a smartphone with internet access are required to use PETRA. This implies that PETRA is currently unavailable in other languages.

### Comparisons With Prior Studies

Thus far, EMA software development has mainly focused on researchers [91-94], who have different needs from clients and clinicians. However, there are three known EMA platforms for clinical purposes: MindLogger [94], m-Path [95], and PsyMate [96,97]. MindLogger does not solely focus on EMA but also on other self-monitoring techniques and mobile interventions. As m-Path and PsyMate are most similar to PETRA, we will briefly review them here. Both platforms offer a web environment for clinicians and clients and a smartphone app that delivers EMA diaries to clients. PsyMate provides a standardized EMA diary: a set list of EMA items (which may differ slightly according to the target group) and 10 assessments per day on a semirandom schedule. m-Path is an open-source platform that targets both researchers and clinicians. It offers full freedom with creating a diary but no decision aid, requiring knowledge of constructing EMA diaries. Similar to PETRA, PsyMate and m-Path were developed based on best practices for EMA and GDPR compliance. Their smartphone apps enable client access to EMA data, which PETRA is still developing. PETRA differs in that it was codeveloped with clients and clinicians from the beginning, balancing personalization with user-friendly diary construction and feedback. Furthermore, the PETRA decision aid and feedback module were specifically developed for use as shared decision-making tools, integrating EMA with contextual information. Finally, its integration into EHR systems via RoQua [38] ensures that PETRA is sustainably embedded in clinical practice. As such, each platform has a different focus, and its concurrent development can advance our understanding of how to optimally implement EMA in clinical practice.

Qualitative work demonstrates that clients and clinicians consider personalized EMA a useful add-on tool for diagnosis and treatment [22-24,78,98]. Similarly, self-monitoring of symptoms is considered safe and acceptable in diverse psychiatric populations [32,99,100]. Our results suggest the need for a collaborative approach to integrating personalized diaries in care, where clients and clinicians decide together on the focus of the EMA and the interpretation of feedback [22]. This corresponds to research demonstrating the importance of shared decision-making for psychiatric outcomes [10,101-103]. Throughout the development process, we realized the importance of tailoring PETRA’s EMA diaries and feedback to diverse clinical settings and client populations. However, much is unknown regarding the optimal type and timing of EMA feedback and how this potentially varies across diagnoses and treatment strategies. This means that PETRA will need to be continuously re-evaluated and adapted based on the diverse needs of clients and clinicians in psychiatric care. This iterative process has also been highlighted in other recent publications on the co-design of self-monitoring technology with users [42,97,104].

### Ethical Considerations

Several ethical considerations arise when developing tools for highly personalized self-monitoring. First, the (sometimes qualitative) data are sensitive, and the privacy of the client should be well protected [105]. For PETRA, software and data transmission security are guaranteed by RoQua [38] and are in compliance with the GDPR. Second, clients may expect that the self-monitoring application alerts them or their clinician in the case of elevated EMA scores, which PETRA currently does not provide. Research indicates that this feature is desired by clients [23]; however, scientific evidence is lacking on how to provide EMA-based alerts with adequate sensitivity and specificity. Therefore, it is important to manage the expectations of clients and clinicians through training. Third, self-monitoring can be burdensome: clients can feel disturbed in daily activities or even experience symptom worsening [22,106]. Clients and clinicians will have to decide together whether the benefits of EMA outweigh this burden. The PETRA application helps clients and clinicians with this decision by providing a burden indicator, which helps them make informed decisions on the number of items. Similarly, the burden may be reduced by placing equal emphasis on symptoms and strengths in personalized diaries, in line with other studies on self-monitoring technology [104,107].

Finally, research suggests that clinician training and support via a helpdesk are important requirements for the implementation of EMA in clinical settings [25,97]. The clinicians in this study also expressed these needs. Training is important to minimize potential health risks and help clinicians construct valid diaries, accurately interpret EMA feedback, and integrate EMA into treatment. Therefore, in the PETRA project, clinicians receive training on the valid use of EMA and the integration of PETRA in treatment, and they have access to a helpdesk. Clinicians are made aware that the feedback should be treated as an additional perspective that is equally (not more) valuable compared with the perspectives of clients and clinicians themselves. Furthermore, short instructive videos are included in PETRA’s various steps, explaining (1) how the decision aid works, (2) what clients should know when completing a diary, and (3) how to interpret the feedback [49].

### Directions for Future Research

Although self-monitoring is already a prominent feature in several treatment modalities and protocols [14,108], an important next step is to determine the clinical effectiveness of adding personalized EMA to the treatment. This is especially relevant as only standardized diaries have been investigated in randomized controlled trials [26,27,29-31]. Extensive personalization options are clearly desired in clinical practice but may mean that effective components may differ across clients, which may complicate research into the effectiveness of personalized EMA [105]. Furthermore, future trials should not only solely focus on symptom improvement but also on more proximate outcomes relevant to the mechanisms of EMA, such as self-management and the therapeutic alliance [52]. Finally, future research will have to investigate the clinical populations and treatment settings for which personalized EMA can be effective. PETRA enables research on these questions. Within the PETRA project, the effectiveness of personalized EMA is tested both qualitatively and quantitatively. PETRA is currently being tested in pilot sessions with clients and clinicians and is continuously updated. Analytics are collected to assess which features of PETRA are used and how, when, and by whom. Furthermore, clients and clinicians are invited to fill out questionnaires on their positive and negative experiences with PETRA, as well as their effects on self-management [109,110] and the therapeutic alliance [111]. This will allow us to examine how personalized EMA influences psychiatric care and to improve PETRA accordingly. These steps can help to set personalized EMA, such as PETRA, as an eHealth technology apart from the myriad of apps available to clients and clinicians, who will be more inclined to use EMA if it is supported by scientific evidence [112].

The second step concerns further scientific validation of the EMA items [113,114] and the development of more innovative EMA feedback. Such endeavors should explore more descriptive visualizations [115] and further develop statistical methods to analyze EMA data. Previous publications on EMA have often highlighted the potential of person-specific statistical models for informing treatment. These models include, for example, network analysis to demonstrate associations between symptoms [54,116,117], early warning signals to alert clients to impending relapse [118,119], and machine learning to differentiate between diagnoses [120]. However, since then, several publications have shown that the outcomes of these models depend on methodological and statistical choices [73] and that researchers disagree on their implications for treatment [74]. Therefore, PETRA focuses on descriptive feedback, with a large role for qualitative descriptions until more consensus on statistical models has been reached.

### Conclusions

PETRA was developed to meet the demands of clients and clinicians for a personalized and user-friendly EMA tool embedded in routine psychiatric care. By collaboratively constructing EMA diaries and interpreting the resulting dynamic feedback, PETRA offers clients and clinicians a new tool to illuminate daily life processes that worsen or alleviate mental health problems. This approach may have beneficial effects on client self-management and the therapeutic alliance and, thus, has the potential to significantly improve clinical care. Our findings demonstrate the importance of a multidisciplinary approach to the development of personalized EMA tools, including clients, clinicians, researchers, and software developers, to ensure that their needs are sufficiently addressed in the design of the tool. PETRA is unique in its codevelopment process, extensive but user-friendly personalization options, integration into EHR systems, transdiagnostic focus, and strong scientific foundation in the design of EMA diaries and feedback. As such, PETRA paves the way for a systematic investigation of the effect of personalized EMA on specialized mental health care.

## Appendix

Multimedia Appendix 1 Supplementary Materials.

## Abbreviations

CeHRes: Center for eHealth Research
EHR: electronic health record
EMA: ecological momentary assessment
GDPR: General Data Protection Regulation
PETRA: Personalized Treatment by Real-time Assessment
RGOc: Rob Giel Research Center
UX: user experience
