# Supplementary material for: A Web-Based Application for Personalized Ecological Momentary Assessment in Psychiatric Care: User-Centered Development of the PETRA Application
Source: JMIR Ment Health. 2022 Aug 9;9(8):e36430. doi: 10.2196/36430 (PMC9399881; doi:10.2196/36430)
Supplement: Multimedia Appendix 1 [file mental_v9i8e36430_app1.pdf]

SUPPLEMENTARY MATERIALS FOR:

PErsonalized Treatment by Real-time Assessment (PETRA): User-centered development of a web-application for personalized diaries in psychiatric care

Fionneke M. Bos, PhD*[1,2], Lino von Klipstein, MSc[2], Ando C. Emerencia[3], PhD, Erwin Veermans, MSc[2], Tom Verhage[2], Evelien Snippe, PhD[2], Bennard Doornbos, MD PhD[4], Grietje Hadders-Prins[1], Marieke Wichers, PhD[2], Harriëtte Riese, PhD[2]

[1] University of Groningen, University Medical Center Groningen, Department of Psychiatry, Groningen, Rob Giel Research Center, The Netherlands
[2] University of Groningen, University Medical Center Groningen, Department of Psychiatry, Interdisciplinary Center Psychopathology and Emotion regulation (ICPE), Groningen, The Netherlands
[3] University of Groningen, Research Support, Faculty of Behavioural and Social Sciences, Groningen, The Netherlands
[4] Lentis Research, Groningen, The Netherlands

*Corresponding author:
Fionneke M. Bos, PhD, University of Groningen, University Medical Centre Groningen, Department of Psychiatry, PO Box 30.001, 9700 RB, Groningen, The Netherlands. Phone: +31 50 361 4860, e-mail: f.m.bos01@umcg.nl.

Contents

Brief introduction to the Supplementary Materials 3

S1. Interview guides for the focus groups, interviews, and usability sessions 4

S2. Quotes of clients and clinicians on the requirements for a web-based EMA tool 7

S3. Overview of goals, subgoals, the matching diary schedule, and diary constructs 9

S4. Further specifications of the diary schedules 11

S5. Matching symptom profiles with diary constructs 12

S6. Burden indicator 13

S7. Features of the decision aid and feedback module 15

S8. Design of the feedback module 16

S9. EMA diary items 18

Semi-random beep schedule 5, 7, or 10 times per day 20

Fixed beep schedule 3, 5, 7, or 10 times per day 37

Once-per-day evening schedule 54

Once-per-day morning schedule 61

References 63

Brief introduction to the Supplementary Materials

In the Supplementary Materials, we will fully share all material that we have developed for PETRA, in the hope this aids other researchers and software platforms in their efforts to bring EMA to clinical practice.

S1 and S2 provide more background for the results of Phase 1-2. S3-S9 provide more details on the various components of the decision aid, EMA schedules, and feedback module of PETRA.

Like explained in the main manuscript, there are four possible EMA schedules in PETRA:

1. **Semi-random schedule** (5, 7 or 10 assessments per day). In this schedule, participants are prompted in semi-random assessment blocks spread out over the day.
2. **Fixed schedule** (3, 5, 7 or 10 assessments per day). In this schedule, participants are prompted at fixed moments throughout the day.
3. **Once-per-day evening schedule**. In this schedule, participants are prompted only once each day, in the evening.
4. **Once-per-day morning schedule**. In this schedule, participants are prompted only once each day, in the morning.

The choices for each schedule depend on the personal 1) goal of the EMA, 2) symptom profile, and 3) perceived burden of each client. In the next pages, we outline the preferred EMA schedules for each goal and symptom profile. We also describe the relevant diary constructs for each goal and symptom profile, and provide the background for the burden indicator. We further share how specific needs of clients and clinicians were translated into specific features of the decision aid and feedback module. Finally, we provide our full EMA item sets for each diary schedule.

S1. Interview guides for the focus groups, interviews, and usability sessions

Phase 1-2: identifying requirements of clients and clinicians
The data gathered in this phase belonged to one of two studies [1, 2].

The interview guide below was previously published in [1]. Clients and clinicians were asked to reflect on the use of EMA in clinical care in interviews and focus groups, and offer suggestions on how EMA could optimally be implemented.

Interview guide for clients and clinicians.

Question	Specific prompts
1. What do you think of EMA?	
2. To what extent would you use EMA yourself?	How?
3. What could be possible consequences of using EMA?	
4. Do you see possible risks or downsides to EMA?	
5. *Patients*: do you have an example of when you would use EMA yourself?<br>*Clinicians*: do you have an example of a patient where you could use EMA?	
6. *Clinicians*: are there patients where you would decide against using EMA?	What kind of patients? Why?
7. *Patients*: would the way you get mental health care change through EMA?<br>*Clinicians*: would the way you give mental health care change through EMA?	How?
8. How do you view the implementation of EMA in mental health care?	Could you identify pitfalls?
9. What would you want to do with EMA-derived feedback?	
10. *Patients:* How would you want to receive EMA-derived feedback?<br>*Clinicians:* How would you discuss the EMA-derived feedback?	*Patients:* Do you discuss it with your clinician or not? How?
11. What kind of questions would you want to ask in the EMA-diaries?	
12. What kind of clinical questions could you answer with EMA?	

Note: examples of generic prompts: "what does […] mean for you?", "can you elaborate?", "what do you mean by […]?", "can you give an example of […]?"

The second interview guide was previously published as well [2]. In this study, clients with bipolar disorder participated in an EMA protocol for four months, and received five EMA assessments per day. Clients and their clinicians were then interviewed on their experiences with adding EMA to their treatment.

Interview guide for clients.

Question	Specific prompts
1. What was it like to participate in this study? How do you reflect on the study?	
2. If I would begin EMA myself, how would you explain it to me?	
3. When would you recommend EMA?	
4. If you could design the EMA and EMA-feedback yourself, with total freedom, how would you do that?	What would you change? What would stay the same?
5. What was your experience with filling in the EMA diaries?	Can you recall positive experiences? Negative experiences?
6. What was your experience with the EMA-feedback?	What was your experience with discussing the feedback with your clinician?
7. Was your participation in the study beneficial in any way?	How?
8. Did your participation change anything in your daily life?	
9. If you would not have participated in this study, what would you have missed?	
10. Did the EMA-feedback affect your treatment?	
11. What would you say if we would include EMA standard in treatment?	

Note: examples of generic prompts: “what does […] mean for you?”, “can you elaborate?”, “what do you mean by […]?”, “can you give an example of […]?”

Interview guide for clinicians.

Question	Specific prompts
1. What was it like to participate in this study as a clinician? How do you reflect on the study?	
2. When would you recommend EMA?	
3. If you could design the EMA and EMA-feedback yourself, with total freedom, how would you do that?	What would you change? What would stay the same?
4. What was your experience with the EMA-feedback?	What was your experience with discussing the feedback with your client?
5. Was the EMA-feedback beneficial to you in any way?	How?
6. Was the EMA-feedback beneficial to your client in any way?	How?
7. Did the EMA-feedback affect the way you provided treatment to the client?	How?
8. If you would not have participated in this study, what would you have missed?	
9. What would you say if we would include EMA standard in treatment?	

Note: examples of generic prompts: “what does […] mean for you?”, “can you elaborate?”, “what do you mean by […]?”, “can you give an example of […]?”

Phase 3: development of PETRA prototypes

Finally, we included the interview guides for the usability sessions with clients and clinicians. These sessions were more structured according to goals and associated questions, which are outlined below.

Interview guide for usability sessions with clients and clinicians.

Goal	Specific questions
1. Gain a shared understanding of what EMA is and what the added benefit could be for clinical practice	Can you tell us what you know about personalized EMA diaries? What is your experience with it?
2. Introduce participants to the goal of PETRA: to implement personalized EMA diaries in clinical practice	What is your first response?
3. Understand the expectations of participants for PETRA	What do you expect of PETRA? Do you see added value of PETRA, for yourself or the other (client or clinician)? Is PETRA always usable, or only in certain stages of care? Can you identify pitfalls or areas that require our attention?
4. Gain feedback on prototypes of PETRA. We walk through PETRA prototypes together. For each page, we ask several questions.	What do you see on the screen? What are your thoughts? What would you click on? What do you miss? What would you have expected to see here?
5. Evaluate the PETRA prototypes in relation to daily clinical practice	Does PETRA meet your expectations? What is your general feeling, positive or negative? What should be changed to improve your evaluation? Would you use EMA in your daily clinical practice? Why (not)?
6. Final thoughts and further steps	Do you have final thoughts? Do you want to be kept informed on our progress? Can we approach you again in the future for a user session?

S2. Quotes of clients and clinicians on the requirements for a web-based EMA tool

Selected (translated) quotes from clients and clinicians on the requirements of a web-based EMA tool.

Requirement	Clients	Clinicians
**Personalization**	ID11: Yes, I don't think it should be a standard item set, but that it should fit with your treatment. It's important to bring that in line with each other.<br>ID13: A bit custom-made, right.<br>ID10: Yes, exactly.	ID34: Maybe you should be able to remove certain items because that is not relevant to that person. I can imagine that. That you make a selection, select items, like 'that should be in there, that shouldn't be in there'.
	ID11: But also that, at a certain point… There's that item, "I feel anxious", I think. Well, I've you've been treated for that, and the anxiety is gone, then you should be able to say 'I'll remove an item from my diary'. That saves another three questions.	ID41: That you can add, every week, if you see people recover from the depression, that you can add some elements every week. So you say: 'can you go try swimming again this week, once or twice this week, and that you then add that element to the assessment. And then after a week, you can say 'right, the swimming went well, let's continue that. What else can you do this week?' And then add another element to the assessment.
**Resource-efficiency**	ID1: Well, I think that if you could, together with your clinician, select some items to fill out, that you can choose for the most important ones, select them from a list. What kind of topics are there to select from? Sleep, activities, mood, and that you choose for yourself, which items you find important and you want to measure.	ID24: It would be helpful if we would have a list to select from, a bit like a menu. That would really lower the threshold to use it, you only have to click. […] And that you have a sort of construction where you are led through a menu of questions. It should look attractive.
	ID14: Because, well, I'm constantly thinking about that psychiatrist that has to work through so many things before you arrive for the session. I just don't see that happening. So, it should be really easy to use and straightforward, so that you can easily see, hey this jumps out, this is something to talk about in the session.	ID32: It should really be ready to use. I shouldn't have to do a lot with it, this sounds really lazy. But all information should be in there, all items should be in there, there should be a fully functional app, and I should be instructed in how it works.

**According to best practices on EMA**	ID2: There is also a danger in there, because, for example, it has been shown that exercise, especially outside, is really important for depressed people. And if I don't find that important at all, then I won't add that [to the assessment], whereas it is proven, over time, that if I have walked in the morning, I feel less depressed in the afternoon. And those are really important things that shouldn't be missed.	ID25: Yes but it really is very strongly a case of garbage in, garbage out, so if you put rubbish in…<br>ID24: You get rubbish out.<br>ID25: You get rubbish out, and then you either see nothing, or you see things that are not right. So you have to carefully define what you are putting in before you put someone to work with it. And potentially it might not have any effect or even adverse effects. But I don't think anybody knows that.
**Privacy**	ID13: Maybe you should also give permission [to share your data with the clinician]. Because otherwise you will have problem with data security.	ID42: There is also a privacy issue, right? Because you carry the phone on you all the time. The more you think about user-friendliness, the more you should consider privacy.

S3. Overview of goals, subgoals, the matching diary schedule, and diary constructs

Goal	Subgoal	Possible EMA schedules (preferred)	Number of assessments per day (preferred)	Preselected diary constructs
**Discover when I experience symptoms**	How do my symptoms vary over time?	Semi-random or fixed	3, 5, 7, 10	
	At what moments of the day or week do I experience symptoms?	Semi-random or fixed	3, 5, 7, 10	
	During what kind of activities do I experience symptoms?	Semi-random	5, 7, 10	- Activities<br>- Social company<br>- Surroundings
	What kind of events influence my symptoms?	Semi-random or fixed	3, 5, 7, 10	- Events
**Discover what influences my symptoms**	How do sleep and lifestyle influence my symptoms?	Semi-random or fixed	3, 5, 7, 10	- Sleep<br>- Food intake<br>- Activities
	What triggers my symptoms?	Semi-random or fixed	3, 5, 7, 10	- Activities<br>- Events
	What helps me cope with my symptoms?	Semi-random or fixed	3, 5, 7, 10	- Activities<br>- Social company<br>- Resilience<br>- Events
	How do my thoughts influence my symptoms?	Semi-random or fixed	3, 5, 7, 10	- Worrying<br>- Self-esteem<br>- Events
	How do my physical and psychological symptoms influence each other?	Semi-random or fixed	3, 5, 7, 10	- Bodily complaints
**Monitoring changes in symptoms**	What is the long-term course of my symptoms?	Fixed, once-per-day, or semi-random	1, 3, 5, 7, 10	
	How do my symptoms change over the course of treatment?	Fixed, once-per-day, or semi-random	1, 3, 5, 7, 10	

	What are the short-term effects of medication?	Semi-random	5, 7, 10	- Medication and side effects

Note: The decision aid brief explains why each schedule is preferred:

- **Brief motivation when semi-random schedule is preferred**

You selected a goal for which it is important to gain a complete picture of what you feel and experience. This can vary strongly from moment to moment. That is why we prefer to assess this at different times during the day. Precisely because you cannot predict the exact timing of the assessments, you gain the best insights into your experiences. Some people do not like this unpredictability. If that is the case for you, you can opt for assessments at fixed time points. But keep in mind that the diaries will offer a less reliable picture of your experiences.

- **Brief motivation when fixed schedule is preferred**

You selected a goal for which it is important to monitor how you feel for a longer period. For example, several weeks or months during your treatment. Most people prefer to complete the diaries at fixed moments during the day.

S4. Further specifications of the diary schedules

Overview of the EMA diary schedules and their specifications

Schedule	Number of assessments per day	Minimal duration of the diary period	Time span each day	Duration of each assessment block	Minimal time in between assessments	Maximum time to complete assessments
**Semi-random**	10	7 days	15h	90 minutes	30 minutes	30 minutes
	7	14 days	15h	15h/7 blocks = 128.57 minutes	30 minutes	30 minutes
	5	14 days	15h	10h/5 blocks = 120 minutes	30 minutes	30 minutes
	3 assessments is **not** possible for a semi-random schedule					
**Fixed**	10	7 days	15h	-	Every 90 minutes	30 minutes
	7	14 days	14h	-	Every 2 hours	30 minutes
	5	14 days	12h	-	Every 3 hours	30 minutes
	3	30 days	12h	-	Every 6 hours	30 minutes
**Once-per-day (evening or morning)**	1	30 days	24h	-	Every 24 hours	180 minutes

Note. Brief explainer

A semi-random EMA schedule consists of several assessment blocks, within which a beep (i.e. a text message with the request to fill in the EMA assessment) will occur at a semi-random moment. A semi-random schedule is helpful when the assessments need to occur at varying time-points from day to day, but also need to be relatively evenly spread out throughout the day. For example, a semi-random schedule with 10 assessments per day will have 10 assessments blocks spread-out over 15 hours, resulting in 10 assessment blocks of 90 minutes each. Within each block, an assessment will occur at a random moment, that is within the block 09.00h-10.30h, at 10.05h. The moment that each assessment occurs thus varies per assessment block and per day. To make sure that assessments do not occur too closely together, we specified a minimal time in between assessments of 30 minutes. This means that assessments occur at least 30 minutes apart, and at most 180 minutes (in case the first assessment occurs in the beginning of the first block, and the second assessment occurs in the end of the second block).

A fixed EMA schedule may be preferred when burden for clients need to minimized. In this schedule, beeps will occur at prespecified times that are consistent across days. For

example, for a fixed schedule with 10 beeps, beeps will occur exactly 90 minutes apart, that is each day at 9:00, then at 10:30, etc.

S5. Matching symptom profiles with diary constructs

Overview of symptom profiles and related diary constructs and items.

Symptom profile	Preselected diary constructs semi-random or fixed schedules	Preselected diary constructs once-per-day schedules	Main item in feedback module
**Anxiety**	Generalized anxiety	Anxiety	At this moment, I feel anxious
**Depression**	Reduced interest and initiative<br>Worrying<br>Self-esteem	Reduced interest and initiative<br>Worrying<br>Self-esteem	At this moment, I feel down
**(Hypo)mania**	(Hypo)mania<br>Sleep<br>Agitation	(Hypo)mania<br>Sleep<br>Agitation	At this moment, I feel agitated
**Psychosis**	Hearing voices<br>Delusions<br>Social company	Psychosis	Since the previous assessment, I was bothered by the voices<br>At this moment, I feel suspicious
**Stress**	Distress and agitation<br>Overstimulation<br>Stress	Distress and agitation<br>Overstimulation<br>Stress	At this moment, I feel stressed
**Something else**	-	-	-

Note: when 'something else' is selected as symptom profile, no diary constructs and diary items are preselected by PETRA.

S6. Burden indicator

The PETRA decision aid contains a ‘burden bar’, which dynamically visualizes the number of items that are selected for a personalized EMA diary. This provides an index of the expected burden for the client, and by extension the clinician, who has to interpret multiple items in the PETRA feedbackmodule. PETRA’s decision aid offers extensive possibilities for personalization; each schedule offers 18-128 items. We therefore want to aid clients and clinicians in finding a balance between gaining additional information and reducing the burden. The burden indicator is thus primarily intended to remind clients and clinician to remain conscious of participant burden and to actively discuss this when compiling the EMA diary.

Currently, research on the effect of the number of items on compliance and experienced burden offers no clear-cut guidelines [3]. Therefore, the PETRA burden indicator is based on our experience with designing EMA studies and will be updated based on user experiences and emerging research. It is likely that the number of items and associated burden differs between and even within individuals, across populations, treatment stages, EMA schedules, and in clinical versus research settings. A recent meta-analysis reported that the number of items did not significantly impact compliance and retention [4]. An empirical study in students reported that 60 EMA items per diary was perceived as more burdensome than 30 EMA items [5]. A qualitative study that compiled researcher experiences with designing EMA studies reported that on average, EMA diaries consist of 30 items [6]. Finally, an overview paper recommended a maximum duration of 2-3 minutes spent per diary [7]. In line with these studies, it is our experience with EMA studies that an average of 25 EMA items is usually perceived as doable by most clients. The additional categories were based on this number.

Example of the burden indicator [translation: 26 items selected (quite long)].

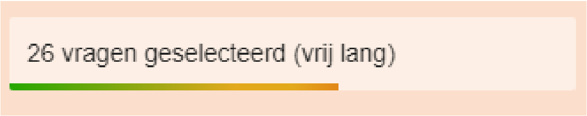

Burden indicator for semi-random and fixed schedules

Number of diary items	Color	Label
10 or fewer	Green	Low
11-25	Green	Average
26-35	Orange	Quite long
36-44	Red	Too long
45 or more	Indicator is red and full	Too long

Burden indicator for once-per-day schedules

Number of diary items	Color	Label
15 or fewer	Green	Low
16-30	Green	Average

31-45	Orange	Quite long
45-60	Red	Too long
60 or more	Indicator is red and full	Too long

S7. Features of the decision aid and feedback module

The focus groups and interviews, as well as the usability sessions, indicated several needs of clients and clinicians that were then translated into specific PETRA features. It is beyond the scope of this paper to list them all, but for clarity we have listed some examples here.

Overview of needs of clients and clinicians and the resulting feature.

**Need**	**Designed feature**
Clients and clinicians want to know exactly how many steps it takes to construct a diary, and what their progress is	Menu: displays the three steps of constructing the diary, as well as the choices already made
Clients and clinicians want to gain a quick overview of the items currently selected in the diary	Preview function: displays the diary exactly as the client views it on the phone
Clients and clinicians want to be able to formulate highly person-specific items themselves	Option to type in your own question. Instructions are provided on how to formulate a valid EMA-item
Clients and clinicians are insecure about how many items per diary is doable	Burden indicator: visualizes the number of items and associated burden
Clinicians want to be able to quickly repeat diaries, which saves time, especially if only a few adaptations are made (e.g., clients want to drop or add certain items or adjust diary frequency)	Repeat function: sets the diary specifications (e.g., selected items, assessment frequency, duration) exactly as a previous diary, which clients and clinicians can then adapt
Clinicians want to save the main conclusions on the diary results in a report, to be able to review the progress in future sessions	Report function: saves current specification of the graph, as well as typed in notes by client or clinician, to the electronic health record

S8. Design of the feedback module

During the focus groups and user sessions, clients and clinicians highlighted several important goals for EMA feedback. These goals were grouped in three main themes, and resulted in different feedback types that were integrated in the feedback module of PETRA.

As outlined in the table below, clients and clinicians mostly agreed on relevant feedback types. An interesting challenge that arose was not necessarily the difference between clients and clinicians, but within-group variability. For example, clinicians with little EMA experience expressed the need for more straightforward and standardized feedback, whereas those with more EMA experience preferred more extensive customization of the EMA diaries and EMA feedback. A similar discrepancy emerged from the sessions with clients as well. Together with the user experience designer, we therefore decided it to be helpful if the feedback module first provides an overview picture of a given topic (e.g., mood, activities). Clients and clinicians can then zoom into moments of interest to get more details. This also makes sure that clients and clinicians are not overwhelmed with all information at once, but rather have the option to go into more detail if desired. Therefore, all the graphs are interactive and customizable.

Main theme	Goal	Desired by	Feedback type
**Fluctuations and changes in symptoms and strengths over time**	Gaining insight into the client's wellbeing	Clients and clinicians	Line graph depicting fluctuations in symptoms/strengths
	Gaining insight into the fluctuations of mood and symptoms	Clients and clinicians	Line graph depicting fluctuations in symptoms/strengths
	What are strengths? What is going well?	Clients	Bar graph depicting average mood, symptoms, and strengths during different (social) contexts)
	Providing a reminder on how the client felt in the previous week, what kind of events they experienced, and what kind of activities they participated in	Clients	Summary blocks demonstrating on which day the client felt best, how often the client was active/passive/social, and sleep duration
**Associations between (social) contexts and symptoms and strengths**	Gaining insight into lifestyle: sleep quality and duration, physical activity, healthy eating, daily structure/rhythm	Clients and clinicians	Summary blocks demonstrating on which day the client felt best, how often the client was active/passive/social, and sleep duration

	Gaining insight into effects and changes, for example due to behavior (smoking, exercising), taking medication, social interactions, (un)healthy eating, addictions	Clients and clinicians	Line graph depicting fluctuations in symptoms/strengths, with a context bar that offers contextual information for specific moments<br><br>Bar graph depicting average mood, symptoms, and strengths during different (social) contexts)
**The impact of (un)pleasant events**	Gaining insight into person-specific triggers: thoughts, events, coping strategies, and the perceived impact of the trigger	Clients	Line graph depicting fluctuations in symptoms/strengths, with a context bar that offers contextual information for specific moments<br>Word cloud that summarizes the text and provides context to thoughts and triggers

S9. EMA diary items

Brief explanation
The following pages will contain all EMA diary items incorporated in PETRA. Like explained in the main manuscript, there are four possible EMA schedules in PETRA:

1. **Semi-random schedule** (5, 7 or 10 assessments per day). In this schedule, participants are prompted in semi-random time-blocks spread out over the day.
2. **Fixed schedule** (3, 5, 7 or 10 assessments per day). In this schedule, participants are prompted at fixed moments throughout the day.
3. **Once-per-day evening schedule**. In this schedule, participants are prompted only once each day, in the evening.
4. **Once-per-day morning schedule**. In this schedule, participants are prompted only once each day, in the morning.

Each EMA schedule has its own item list.
In the **semi-random and fixed schedules**, the following constructs are addressed:

1. Mood
2. (Hypo)mania
3. Agitation and stress
4. Anxiety
5. Psychosis
6. Bodily complaints
7. Reduced interest and initiative
8. Surroundings, behavior, agents
9. Social interactions
10. Worrying
11. Self-esteem
12. Positivity
13. Formulate your own questions

In the **once-per-day schedules**, the following constructs are addressed:

1. Mood of the day
2. Mood of the morning
3. (Hypo)mania
4. Agitation and stress
5. Anxiety
6. Psychosis
7. Bodily complaints
8. Reduced interest and initiative
9. Events
10. Worrying
11. Self-esteem
12. Functioning and resilience
13. Sleep during the day
14. Sleep at night
15. Formulate your own questions

Note that some constructs contain multiple subconstructs.

Translation

All EMA items are originally in Dutch, but were translated to English for the purposes of this paper.

Response scale
Most items were answered on a 0-100 visual analogue scale (VAS). Other response options are outlined.

Item ordering
The EMA diary items are ordered differently in the decision aid versus in the actual diary that the participants complete on their smartphones. In the decision aid, all items are grouped in constructs (as presented in the EMA item sets below). Some items (e.g., "I feel stressed") are relevant to multiple constructs and are therefore offered multiple times in the decision aid.

After all diary items are compiled in the decision aid, PETRA will remove duplicate items and reorder the items to form the diary for the participant. In this diary, all EMA items are ordered as follows:

1. In-the-moment mood items (e.g., "Right now, I feel cheerful")
2. In-the-moment other items (e.g., "Right now, my thoughts are racing")
3. In-the-moment contextual items (e.g., "Right now, what am I doing?")
4. Retrospective items (e.g., "Since the previous measurement, I have had fun")
5. Personally formulated items
6. Qualitative item ("If you would like to note down something else about the period since the previous measurement, you can do that here."

Overlap in schedules
The semi-random and fixed EMA schedules are very similar. In the fixed schedule, a few EMA items were excluded or adapted from 'in-the-moment' to retrospective items, to be able to capture the momentary experience.

The once-per-day schedules often are an insightful addition to the semi-random and fixed EMA schedules. Therefore, the PETRA decision aid has integrated some of the items of the once-per-day-schedules in the relevant (sub)constructs of the more frequent EMA schedules. These items are marked by * in the once-per-day schedules.

Match with (sub)goals or symptom profiles
Green highlighted items are automatically selected if it is matched to a (sub)goal or symptom profile. For example, selecting the profile "Anxiety" automatically selects the subconstruct "Anxiety", which selects three out of five EMA items of this subconstruct. These highlighted items were chosen because they were considered relevant for a large group of patients.

References
For each item, we have included a (non-exhaustive) reference list of published EMA studies that have used these EMA items in diverse populations. In the fixed and semi-random schedules, 74% of the items originated from previous EMA research, and 26% were newly developed based on suggestions by clients and clinicians. For the once-per-day evening schedule, 92% items stemmed from previous EMA research, and 8% were newly developed.

Semi-random beep schedule 5, 7, or 10 times per day

Constructs, subconstructs, and EMA items

Main construct: Mood						
**Subconstruct**	**EMA item (NL)**	**EMA item (EN)**	**Response scale (NL)**	**Response scale (EN)**	**Construct**	**Reference**
**Mood (obligatory)**	Op dit moment voel ik me opgewekt	Right now, I feel cheerful	Helemaal niet - heel erg	Not at all - very much	PA high arousal	[2, 8-12]
	Op dit moment voel ik me somber	Right now, I feel down	Helemaal niet - heel erg	Not at all - very much	NA low arousal	[8-13]
	Op dit moment voel ik me tevreden	Right now, I feel content	Helemaal niet - heel erg	Not at all - very much	PA low arousal	[2, 8-12]
	Op dit moment voel ik me geïrriteerd	Right now, I feel irritated	Helemaal niet - heel erg	Not at all - very much	NA high arousal	[2, 10-13]
	Op dit moment voel ik me energiek	Right now, I feel energetic	Helemaal niet - heel erg	Not at all - very much	PA high arousal	[8-12]
	Op dit moment voel ik me onrustig	Right now, I feel restless	Helemaal niet - heel erg	Not at all - very much	NA high arousal	[8-11]
**Standard last item of the EMA question-naire**	Als u nog iets anders over de periode sinds het vorige meetmoment wilt noteren, dan kunt u dat hier doen.	If you would like to note down something else about the period since the previous measurement, you can do that here.	[String (optioneel), max 280 karakters]	[String (optional), max 280 characters]	Qualitative description of anything of relevance not captured by the other items	
**Main construct: (Hypo)mania**						
**Subconstruct**	**EMA item (NL)**	**EMA item (EN)**	**Response scale (NL)**	**Response scale (EN)**	**Construct**	**Reference**
**(Hypo)mania**	Op dit moment voel ik me bijzonder goed	Right now, I feel extremely well	Helemaal niet - heel erg	Not at all - very much	Expansive mood	[2]
	Op dit moment voel ik me gejaagd	Right now, I feel agitated	Helemaal niet - heel erg	Not at all - very much	Agitation	[2, 14]
	Op dit moment voel ik me prikkelbaar	Right now, I feel irritable	Helemaal niet - heel erg	Not at all - very much	Irritable mood	[9]

	Op dit moment gaan mijn gedachten snel	Right now, my thoughts are racing	Helemaal niet - heel erg	Not at all - very much	Racing thoughts	[2, 14]
	Op dit moment ben ik snel afgeleid	Right now, I am easily distracted	Helemaal niet - heel erg	Not at all - very much	Distractability	[2, 9]
	Op dit moment zit ik vol goede ideeën	Right now, I am full of good ideas	Helemaal niet - heel erg	Not at all - very much	Expansive ideas	[2, 15]
	Op dit moment heb ik zin om met anderen af te spreken	Right now, I feel like socializing	Helemaal niet - heel erg	Not at all - very much	Need for social contact	[2]
	Op dit moment kan ik snel schakelen	Right now, I can focus and switch easily	Helemaal niet - heel erg	Not at all - very much	Thinking clearly	[2, 14]
	Sinds het vorige meetmoment heb ik contact gezocht met anderen	Since the previous measurement, I have sought contact with others	Helemaal niet - heel erg	Not at all - very much	Actual social contact	[2]
	Sinds het vorige meetmoment ben ik spraakzaam geweest	Since the previous measurement, I have been talkative	Helemaal niet - heel erg	Not at all - very much	Talkativeness	[2, 14]
			**Main construct: Agitation and stress**			
**Subconstruct**	**EMA item (NL)**	**EMA item (EN)**	**Response scale (NL)**	**Response scale (EN)**	**Construct**	**Reference**
**Agitation**	Op dit moment voel ik me gestrest	Right now, I feel stressed	Helemaal niet - heel erg	Not at all - very much	Stressed feeling	[10]
	Op dit moment voel ik me wantrouwig	Right now, I feel suspicious	Helemaal niet - heel erg	Not at all - very much	Paranoid delusion	[16, 17]
	Op dit moment voel ik me gespannen	Right now, I feel tense	Helemaal niet - heel erg	Not at all - very much	Stressed feeling	[10]
	Op dit moment voel ik me gejaagd	Right now, I feel agitated	Helemaal niet - heel erg	Not at all - very much	Agitation	[2, 14]
	Op dit moment voel ik me prikkelbaar	Right now, I feel irritable	Helemaal niet - heel erg	Not at all - very much	Irritable mood	[9]
**Over-stimulation**	Op dit moment voel ik me overprikkeld	Right now, I feel over-stimulated	Helemaal niet - heel erg	Not at all - very much	Over-stimulation	Adapted from[18]

	Op dit moment is het een chaos in mijn hoofd	Right now, my mind is reeling	Helemaal niet - heel erg	Not at all - very much	Disorganized thinking	[17]
	Op dit moment ben ik snel afgeleid	Right now, I am easily distracted	Helemaal niet - heel erg	Not at all - very much	Concentration/ distractability	[9]
	Op dit moment kan ik me goed concentreren	Right now, I can concentrate well	Helemaal niet - heel erg	Not at all - very much	Concentration	[10, 12, 17]
**Stress**	Op dit moment voel ik me gestrest	Right now, I feel stressed	Helemaal niet - heel erg	Not at all - very much	Stressed feeling	[19]
	Op dit moment zie ik op tegen de rest van de dag	Right now, I am dreading the rest of the day	Helemaal niet - heel erg	Not at all - very much		[10]
	Denk aan de meest plezierige gebeurtenis of activiteit sinds het vorige meetmoment. Hoe plezierig was deze gebeurtenis?	Think of the most pleasant event or activity since the previous measurement. How pleasurable was this event?	Helemaal niet - heel erg plezierig	Not pleasant at all - very pleasant	Reward sensitivity	[11, 12, 19]
	Hoe heftig was deze gebeurtenis?	How intense was this event?	Helemaal niet - heel erg	Not at all - very much	Reward sensitivity	[11]
	De plezierige ervaring had te maken met:	The pleasant event concerned:	[String (optioneel), max 280 karakters]	[String (optional), max 280 characters]	Qualitative description of event	[11]
	Denk aan de meest onplezierige gebeurtenis of activiteit sinds het vorige meetmoment. Hoe onplezierig was deze gebeurtenis?	Think of the most unpleasant event or activity since the previous measurement. How unpleasant was this event?	Helemaal niet onplezierig - heel erg onplezierig	Not unpleasant at all - very unpleasant	Stress reactivity	[11, 12, 19]
	Hoe heftig was deze gebeurtenis?	How intense was this event?	Helemaal niet - heel erg	Not at all - very much	Stress reactivity	[11]

	De onplezierige ervaring had te maken met	The unpleasant event concerned:	[String (optioneel), max 280 karakters]	[String (optional), max 280 characters]	Qualitative description of event	[11]
			**Main construct: Anxiety**			
**Subconstruct**	**EMA item (NL)**	**EMA item (EN)**	**Response scale (NL)**	**Response scale (EN)**	**Construct**	**Reference**
	Op dit moment voel ik me veilig	Right now, I feel safe	Helemaal niet - heel erg	Not at all - very much	Feeling safe	[20]
	Op dit moment voel ik me gespannen	Right now, I feel tense	Helemaal niet - heel erg	Not at all - very much	Stressed feeling	[19]
	Op dit moment ben ik aan het piekeren	Right now, I worry	Helemaal niet - heel erg	Not at all - very much	Worrying	[10, 11]
	Sinds het vorige meetmoment heb ik iets afgezegd of vermeden	Since the previous measurement, I have cancelled or avoided something	Helemaal niet - heel erg	Not at all - very much	Avoidance	[21]
	Op dit moment ben ik bang om alleen te zijn	Right now, I am scared of being alone	Helemaal niet - heel erg	Not at all - very much	Anxiety for being alone	[21]
**Intrusions and compulsive complaints**	Sinds het vorige meetmoment had ik nare herinneringen	Since the previous measurement, I had intrusive memories	Helemaal niet - heel erg	Not at all - very much	Intrusions	[21]
	Sinds het vorige meetmoment heb ik dwangklachten gehad	Since the previous measurement, I had compulsive complaints	Helemaal niet - heel erg	Not at all - very much	Compulsive complaints	[21]
**Panic**	Op dit moment ben ik bang dat ik een paniekaanval krijg	Right now, I am scared of having a panic attack	Helemaal niet - heel erg	Not at all - very much	Anxiety for panic attacks	[21]
	Sinds het vorige meetmoment had ik last van paniekklachten	Since the previous measurement, I had panic complaints (f.e.,	Helemaal niet - heel erg	Not at all - very much	Physical anxiety complaints	[21]

	(bijv. hartkloppingen, zweten, misselijkheid)	heart palpitations, sweating, nausea)				
**Derealization**	Op dit moment voel ik me onwerkelijk	Right now, I feel surreal	Helemaal niet - heel erg	Not at all - very much	Derealization	[21]
	Op dit moment ben ik bang om de controle te verliezen	Right now, I am scared of losing control	Helemaal niet - heel erg	Not at all - very much	Anxiety for losing control	[21]
			**Main construct: Psychosis**			
**Subconstruct**	**EMA item (NL)**	**EMA item (EN)**	**Response scale (NL)**	**Response scale (EN)**	**Construct**	**Reference**
**Hearing voices**	Sinds het vorige meetmoment hoorde ik stemmen	Since the previous measurement, I heard voices	- Ja<br>- Nee	- Yes<br>- No	Hearing voices	[20]
(if yes on first item)	Sinds het vorige meetmoment had ik last van de stemmen	Since the previous measurement, I was bothered by the voices	Helemaal niet - heel erg	Not at all - very much	Bothered by the voices	Adapted from [22]
(if yes on first item)	Wat was de voornaamste copingstrategie die je hebt gebruikt tegen de stemmenlast?	What was your main coping strategy that you used against the burden you experienced by the voices?	**Checklist: (één optie mogelijk)**<br>- Doen wat de stemmen zeggen<br>- In gesprek gaan met de stemmen<br>- De stemmen wegsturen of zeggen te stoppen<br>- Afleiding door iets sociaals<br>- Afleiding door iets taligs (bijv. lezen, zingen)<br>- Afleiding door werkgeheugenbelasting (bijv. TemStem, woordpuzzel)<br>- Blowen of alcohol drinken	**Checklist (one option possible)**<br>- Do what voices say<br>- Talk to voices<br>- Send voices away or order to stop<br>- Distract by doing something social<br>- Distract by something verbal (f.e., reading, singing)<br>- Distract by taxing the working memory (f.e., TemStem, word puzzle)<br>- Using drugs or alcohol<br>- Taking medication	Coping with the voices	Adapted from [22]

			- Medicatie innemen<br>- Iets ontspannens (bijv. sporten, muziek luisteren)<br>- Geruststellende gedachten<br>- Ik gebruikte geen copingstrategie<br>- Iets anders	- Something relaxing (f.e., doing sports, listening to music)<br>- Reassuring thoughts<br>- I did not use a coping strategy<br>- Something else		
(if yes on first item)	In welke mate heeft deze copingstrategie je geholpen de stemmenlast te verminderen?	To what extent has the coping strategy helped you to reduce the burden of the voices?	Helemaal niet - heel erg	Not at all - very much	Effectivity of coping strategies	Adapted from [22]
(if yes on first item)	De stemmen maakten mij boos	The voices made me angry	Helemaal niet - heel erg	Not at all - very much	Emotions due to the voices	Adapted from [22]
(if yes on first item)	De stemmen maakten mij blij	The voices made me happy	Helemaal niet - heel erg	Not at all - very much	Emotions due to the voices	Adapted from [22]
(if yes on first item)	De stemmen maakten mij bedroefd	The voices made me sad	Helemaal niet - heel erg	Not at all - very much	Emotions due to the voices	Adapted from [22]
(if yes on first item)	De stemmen maakten mij beschaamd	The voices made me feel ashamed	Helemaal niet - heel erg	Not at all - very much	Emotions due to the voices	Adapted from [22]
(if yes on first item)	De stemmen maakten mij bang	The voices scared me	Helemaal niet - heel erg	Not at all - very much	Emotions due to the voices	Adapted from [22]
**Delusions**	Op dit moment voel ik me wantrouwig	Right now, I feel suspicious	Helemaal niet - heel erg	Not at all - very much	Paranoid delusion	[13, 17, 20]
	Op dit moment voel ik me veilig	Right now, I feel safe	Helemaal niet - heel erg	Not at all - very much	Paranoid delusion	[20]
	Op dit moment lijkt alles betekenis te hebben	Right now, I feel as if	Helemaal niet - heel erg	Not at all - very much	Verhoogde betekenisgeving	[13]
	Op dit moment voel ik me onwerkelijk	Right now, I feel surreal	Helemaal niet - heel erg	Not at all - very much	Derealization	[21]

	Het is weer bezig	It is going on again	Helemaal niet - heel erg	Not at all - very much	Person-specific delusion	[23]
	Op dit moment worden mijn gedachten door anderen beïnvloed	Right now, my thoughts are influenced by others	Helemaal niet - heel erg	Not at all - very much	Beïnvloedingswaan	[13, 17, 20]
Main construct: Bodily complaints						
**Subconstruct**	**EMA item (NL)**	**EMA item (EN)**	**Response scale (NL)**	**Response scale (EN)**	**Construct**	**Reference**
**Bodily complaints**	Op dit moment heb ik last van:	Right now, I am bothered by:	**Checklist (meerdere opties mogelijk):**<br>- Ademhalingsproblemen (kortademigheid, moeilijk adem kunnen krijgen)<br>- Pijn of drukkend gevoel op de borst<br>- Hartkloppingen<br>- Misselijkheid of maag die van streek is<br>- Verstopping, dunne ontlasting of diarree<br>- Pijn in de buik of maagstreek<br>- Hoofdpijn<br>- Duizeligheid<br>- Vermoeidheid<br>- Rugpijn<br>- Pijn in spieren of gewrichten<br>- Verdoofd of tintelend gevoel	**Checklist (multiple responses possible):**<br>- Trouble breathing (shortness of breath, breathing difficulties)<br>- Pain or pressing feeling on the chest<br>- Heart palpitations<br>- Nausea or upset stomach<br>- Constipation, diarrhea<br>- Abdominal pain or stomachaches<br>- Headache<br>- Dizziness<br>- Fatigue<br>- Back pain<br>- Pain in joints or muscles<br>- Numbness or tingling	Bodily complaints	Adapted from [24]
(for each selected complaint)	Hoeveel had je last van [klacht]	How much were you bothered by [complaint]	Helemaal niet - heel erg	Not at all - very much	Bodily complaints	[25]

	Sinds het vorige meetmoment was ik in gedachten veel met mijn lichamelijke klachten bezig	Since the previous measurement, I was preoccupied by my bodily complaints	Helemaal niet - heel erg	Not at all - very much	Preoccupation with bodily complaints	[25]
	Sinds het vorige meetmoment zocht ik afleiding van mijn lichamelijke klachten	Since the previous measurement, I sought distraction from my bodily complaints	Helemaal niet - heel erg	Not at all - very much	Seeking distraction from bodily complaints	[25]
	Sinds het vorige meetmoment voelde ik mij belemmerd door mijn lichamelijke klachten	Since the previous measurement, I felt hindered by my bodily complaints	Helemaal niet - heel erg	Not at all - very much	Feeling hindered by bodily complaints	[25]
**Main construct: Reduced interest**						
**Subconstruct**	**EMA item (NL)**	**EMA item (EN)**	**Response scale (NL)**	**Response scale (EN)**	**Construct**	**Reference**
**Reduced interest and initiative**	Ik heb zin in de rest van de dag	I look forward to the rest of the day	Helemaal niet - heel erg	Not at all - very much	Anticipatory reward	[2]
	Op dit moment heb ik zin om met anderen te zijn	Right now, I feel like being with others	Helemaal niet - heel erg	Not at all - very much	Need for socializing	[10]
	Op dit moment voel ik me lusteloos	Right now, I feel listless	Helemaal niet - heel erg	Not at all - very much	Anhedonia	[10, 12]
**Main construct: Surroundings, behavior, agents**						
**Subconstruct**	**EMA item (NL)**	**EMA item (EN)**	**Response scale (NL)**	**Response scale (EN)**	**Construct**	**Reference**
**Events**	Denk aan de meest plezierige gebeurtenis of activiteit sinds het vorige meetmoment. Hoe plezierig was dit?	Think of the most pleasant event or activity since the previous measurement. How pleasurable was this?	Helemaal niet - heel erg plezierig	Not at all - very pleasant	Reward sensitivity	[11, 19]

	Hoe heftig was deze gebeurtenis of activiteit?	How intense was this event?	Helemaal niet - heel erg	Not at all - very much	Reward sensitivity	[11]
	De plezierige ervaring had te maken met:	The pleasant event concerned:	[String (optioneel), max 280 karakters]	[String (optional), max 280 characters]	Qualitative description of event	[11, 19]
	Denk aan de meest onplezierige gebeurtenis of activiteit sinds het vorige meetmoment. Hoe onplezierig was dit?	Think of the most unpleasant event or activity since the previous measurement. How unpleasant was this?	Helemaal niet - heel erg onplezierig	Not at all – very unpleasant	Stress reactivity	[11]
	Hoe heftig was dit?	How intense was this?	Helemaal niet - heel erg	Not at all - very much	Stress reactivity	[11]
	De onplezierige gebeurtenis had te maken met	The unpleasant event concerned:	[String (optioneel), max 280 karakters]	[String (optional), max 280 characters]	Qualitative description of event	[11]
**Activity**	Op dit moment, wat doe ik?	Right now, what am I doing?	**Checklist (één optie mogelijk):**<br>- Werken/studeren<br>- Sporten/wandelen/ fietsen<br>- Hobby (bijv. muziek maken, klussen)<br>- Uitje (bijv. naar de stad, concert)<br>- Iets rustigs (bijv. lezen, tv kijken)<br>- Met iemand praten<br>- Iets intiems (bijv. knuffelen, seks)<br>- Whatsappen	**Checklist (one option possible):**<br>- Working/studying<br>- Doing sports/walking/ biking<br>- Hobby (f.e., making music, DIY)<br>- Outing (f.e., going into the city or a concert)<br>- Something relaxing (f.e., reading, watching TV)<br>- Talking to someone<br>- Something intimate (cuddling, sex)<br>- Whatsapp/texting	(social) context	[9-11]

			- Social Media<br>- Eten<br>- Huishouden/ boodschappen/ administratie<br>- Zelfverzorging (bijv. douchen, opmaken)<br>- Rusten/niets<br>- Ik ben onderweg<br>- Iets anders	- Social Media<br>- Eating<br>- Household chores/ grocery shopping/ administration<br>- Self-care (f.e., showering, applying make-up)<br>- Resting/nothing<br>- Travelling, or moving from one place to another<br>- Something else		
	Deze activiteit vind ik…	I find this activity…	Zeer onplezierig - zeer plezierig	Very unpleasant – very pleasant		[9-11]
	Sinds het vorige meetmoment ben ik lichamelijk actief geweest	Since the previous measurement, I have been physically active	Helemaal niet - heel erg	Not at all - very much	Physical activity	[10, 11, 26]
**Surroundings**	Op dit moment ben ik buiten	Right now, I am outside	- Ja<br>- Nee	- Yes<br>- No	Being outside	[21]
(if outside)	Hoeveel daglicht is er?	How much daylight is there?	Helemaal niet – heel veel	Not at all – a lot	Daylight	[21]
	Op dit moment, waar ben ik?	Right now, where am I?	**Checklist (één optie mogelijk):**<br>- Thuis<br>- School/werk<br>- Bij familie/vrienden<br>- Café, bibliotheek, park<br>- Supermarkt/winkel<br>- In de natuur<br>- Hulpverlening<br>- Onderweg	**Checklist (one option possible):**<br>- Home<br>- School/work<br>- Family/friends<br>- Cafe/library/park<br>- Supermarket/shop<br>- Nature<br>- Health facility	Location	[9]

			- Ergens anders	- Travelling, or moving from one place to another<br>- Somewhere else		
**Food, agents, and side-effects**	Sinds het vorige meetmoment heb ik de volgende middelen gebruikt	Since the previous measurement, I have taken the following	**Checklist (meerdere opties mogelijk):**<br>- Geen<br>- Sigaretten<br>- Koffie of energydrank<br>- Alcohol<br>- Snacks<br>- Snoep<br>- Cannabis<br>- Medicatie	**Checklist (multiple responses possible):**<br>- None<br>- Cigarettes<br>- Coffee or energy drink<br>- Alcohol<br>- Snacks<br>- Candy<br>- Cannabis<br>- Medication	Middelen/medic atiegebruik	[17]
	Sinds het vorige meetmoment heb ik deze ervaring(en) gehad	Since the previous measurement, I have had the following experiences	**Checklist (meerdere opties mogelijk):**<br>- Hoofdpijn<br>- Hongergevoel<br>- Misselijkheid<br>- Suf of slaperig<br>- Pijnlijke of stijve spieren<br>- Onrust van binnen<br>- Duizelig<br>- Droge mond<br>- Vlak<br>- Traag<br>- Geen van bovenstaande	**Checklist (multiple responses possible):**<br>- Headache<br>- Hunger<br>- Nausea<br>- Drowsy or sleepy<br>- Painful or stiff muscles<br>- Restlessness<br>- Dizziness<br>- Dry mouth<br>- Flat<br>- Slow<br>- None of the above	Medication side-effects	[21]
**Food**	Sinds het vorige meetmoment heb ik het volgende gegeten	Since the previous measurement, I have eaten the following	**Checklist (meerdere opties mogelijk):**<br>- Broodmaaltijd<br>- Gezond tussendoortje<br>- Warme maaltijd	**Checklist (multiple responses possible):**<br>- Sandwich<br>- Healthy snack<br>- Hot meal	Food type	[21]

			- Snoep<br>- Snacks<br>- Niets	- Candy<br>- Snack<br>- Nothing		
	Sinds het vorige meetmoment heb ik gegeten…	Since the previous measurement, I have eaten…	Veel minder dan normaal - veel meer dan normaal	A lot less than usual – a lot more than usual	Food quantity	[21]
	Sinds het vorige meetmoment heb ik gezond gegeten	Since the previous measurement, I have eaten healthily	Helemaal niet - heel erg	Not at all - very much	Health level of food	[21]
			**Hoofdthema: Social interactions**			
**Subconstruct**	**EMA item (NL)**	**EMA item (EN)**	**Response scale (NL)**	**Response scale (EN)**	**Construct**	**Reference**
**Company**	Op dit moment ben ik…	Right now, I am…	**Checklist (één optie mogelijk):**<br>- Alleen<br>- Alleen met huisdier<br>- In gezelschap	**Checklist (one response possible):**<br>- Alone<br>- Alone with pet<br>- In company	Alone/with others	[11]
(if in company)	Op dit moment, met wie ben ik?	Right now, with whom am I?	**Checklist (meerdere opties mogelijk):**<br>- Partner<br>- Huisgenoten<br>- Gezin<br>- Familie uitwonend<br>- Vrienden<br>- Collega's/ klasgenoten<br>- Hulpverleners<br>- Kennissen<br>- Onbekenden	**Checklist (multiple responses possible):**<br>- Partner<br>- House mates<br>- Family<br>- Extended family<br>- Friends<br>- Colleagues/class mates<br>- Clinicians<br>- Acquaintances<br>- Strangers	Company type	[9, 11, 13]
(if in company)	Vul hier in met wie je bent. *NB: noteer hier geen namen en dergelijke. Denk om privacy.*	Indicate with whom you are. *Note: do not mention names here. Remember privacy.*	[String (optioneel), max 280 karakters]	[String (optional), max 280 characters]		[27-29]

(if in company)	Ik vind dit gezelschap aangenaam	I find this company agreeable	Helemaal niet - heel erg	Not at all - very much	Social stress	[9, 11, 30]
(if in company)	Ik heb het gevoel dat deze mensen me mogen	I feel these people like me	Helemaal niet - heel erg	Not at all - very much	Social stress	[30]
(if alone)	Ik was liever in gezelschap geweest	I'd rather have been in company	Helemaal niet - heel erg	Not at all - very much	Social stress	[13, 30]
(if alone)	Ik vind het fijn om nu alleen te zijn	Right now, I like being alone	Helemaal niet - heel erg	Not at all - very much	Social stress	[30]
(if in company)	Ik voel me geaccepteerd in dit gezelschap	I feel accepted by this company	Helemaal niet - heel erg	Not at all - very much	Social stress	[11, 30]
(if in company)	Ik voel me op mijn gemak in dit gezelschap	I feel at ease in this company	Helemaal niet - heel erg	Not at all - very much	Social stress	[30]
**Connected-ness**	Sinds het vorige meetmoment voelde ik me eenzaam	Since the previous measurement, I have felt lonely	Helemaal niet - heel erg	Not at all - very much	Loneliness	[9, 13]
	Op dit moment voel ik dat ik om anderen geef	Right now, I feel I care for others	Helemaal niet - heel erg	Not at all - very much	Caring for others	[27-29]
	Op dit moment voel ik dat anderen om mij geven	Right now, I feel that others care about me	Helemaal niet - heel erg	Not at all - very much	Feeling others care about you	[17, 27-29]
	Op dit moment voel ik me verbonden met anderen	Right now, I feel connected with others	Helemaal niet - heel erg	Not at all - very much	Feeling connected	[27-29]
**Interactions**	Heb je sinds het vorige meetmoment gesprekken gehad? *Gesprekken duren langer dan 5 minuten. Dit kunnen face-to-face gesprekken of*	Since the previous measurement, did you have interactions? *Interactions last longer than 5 minutes, and can be face-to-face interactions or via online (social) media.*	- Ja<br>- Nee	- Yes<br>- No	Had interactions	[27-29]

	gesprekken via online (sociale) media zijn.					
	Sinds het vorige meetmoment, hoeveel gesprekken heb je gehad?	Since the previous measurement, how many interactions did you have?	[Geef getal aan]	[indicate number]	Number of interactions	[27-29]
	Denk aan het voor jou meest belangrijk gesprek sinds het vorige meetmoment. Met wie heb je dit gesprek gehad? *NB: noteer hier geen namen en dergelijke. Denk om privacy.*	Think about the most important interaction since the previous measurement. With whom did you have this interaction? *Note: do not mention names here. Remember privacy.*	[String (optioneel), max 280 karakters]	[String (optional), max 280 characters]	Interaction partner	[27-29]
	Wat voor gesprek was dit?	What kind of interaction was this?	**Checklist (één optie mogelijk):**<br>- Face-to-face<br>- Telefoongesprek<br>- Videobelgesprek<br>- Gesprek via email of sociale media (bijv. Whatsapp)<br>- Iets anders	**Checklist (one option possible):**<br>- Face-to-face<br>- By phone<br>- By video messaging<br>- By e-mail or social media (f.e., whatsapp)<br>- Something else	Medium	[27-29]
	Hoe belangrijk was dit gesprek voor je?	How important was this interaction for you?	Helemaal niet - heel erg	Not at all - very much	Interaction importance	[27-29]
	Wat maakte dit gesprek belangrijk en waar ging het over?	What made this interaction important and what was it about?	[String (optioneel), max 280 karakters]	[String (optional), max 280 characters]	Qualitative description of interaction importance	[27-29]
	Hoe lang duurde het gesprek?	What was the duration of the interaction?	[Geef getal aan (minuten)]	[Indicate number (minutes)]	Interaction duration	[27-29]

	Hoe (on)plezierig was de inhoud van het gesprek?	How (un)pleasant was the content of the interaction?	Heel erg onplezierig – heel erg plezierig	Very unpleasant – very pleasant	(Un)pleasantnes s of interaction content	[27-29]
	Hoe (on)plezierig heb je het gesprek zelf ervaren? *Denk hierbij bijvoorbeeld aan de houding en toon van je gesprekspartner.*	How (un)pleasant was the interaction itself? *Think about the attitude and tone of your interaction partner.*	Heel erg onplezierig – heel erg plezierig	Very unpleasant – very pleasant	(Un)pleasant-ness of interaction itself	[27-29]
	In hoeverre beïnvloedde het gesprek je stemming?	To what extent did the interaction influence your mood?	Mijn stemming werd veel slechter - mijn stemming werd veel beter	My mood worsened a lot – my mood improved a lot	Impact of interaction on mood	[27-29]
	In hoeverre beïnvloedde het gesprek je energieniveau?	To what extent did the interaction influence your energy level?	Het kostte me veel energie - Het leverde me veel energie op	It cost me a lot of energy – it gave me a lot of energy	Impact of interaction on energy level	[27-29]
	In hoeverre kon je jezelf zijn in dit gesprek?	To what extent could you be yourself in this interaction?	Helemaal niet - heel erg	Not at all - very much	Being yourself	[27-29]
	Ik voelde me gesteund door mijn gesprekspartner(s)	I felt supported by my interaction partner(s).	Helemaal niet - heel erg	Not at all - very much	Feeling supported	[27-29]
	Is er sinds het vorige meetmoment nog een ander gesprek geweest dat je graag wilt beschrijven?	Has there been another interaction since the previous measurement that you would like to describe?	- Ja<br>- Nee<br>[indien ja, herhaal vorige items]	- Yes<br>- No<br>[if yes, repeat previous items]	Another interaction	[27-29]
			**Main construct: Worrying**			
**Subconstruct**	**EMA item (NL)**	**EMA item (EN)**	**Response scale (NL)**	**Response scale (EN)**	**Construct**	**Reference**
**Worrying**	Op dit moment ben ik aan het piekeren	Right now, I am worrying	Helemaal niet - heel erg	Not at all - very much	Worrying	[10, 11]

Subconstruct	EMA item (NL)	EMA item (EN)	Response scale (NL)	Response scale (EN)	Construct	Reference
	Op dit moment laten mijn gedachten me niet los	Right now, I can't disconnect from my thoughts	Helemaal niet - heel erg	Not at all - very much	Worrying	[17]
			**Main construct: Self-esteem**			
**Subconstruct**	**EMA item (NL)**	**EMA item (EN)**	**Response scale (NL)**	**Response scale (EN)**	**Construct**	**Reference**
**Self-esteem**	Op dit moment voel ik me goed over mezelf	Right now, I feel good about myself	Helemaal niet - heel erg	Not at all - very much	Self-esteem	[17]
	Op dit moment zijn mijn gedachten over mezelf…	Right now, my thoughts about myself are…	Heel erg negatief – heel erg positief	Very negative – very positive	Self-esteem	[10]
	Op dit moment heb ik het gevoel te kort te schieten	Right now, I feel inadequate	Helemaal niet - heel erg	Not at all - very much	Self-esteem	[9, 10]
	Op dit moment voel ik me nuttig	Right now, I feel useful	Helemaal niet - heel erg	Not at all - very much	Productivity	[2]
			**Main construct: Positivity**			
**Subconstruct**	**EMA item (NL)**	**EMA item (EN)**	**Response scale (NL)**	**Response scale (EN)**	**Construct**	**Reference**
**Positivity**	Op dit moment voel ik me goed over mezelf	Right now, I feel good about myself	Helemaal niet - heel erg	Not at all - very much	Self-esteem	[21]
	Op dit moment heb ik zin om met anderen te zijn	Right now, I feel like socializing	Helemaal niet - heel erg	Not at all - very much	Need for social contact	[10]
	Op dit moment voel ik me ontspannen	Right now, I feel relaxed	Helemaal niet - heel erg	Not at all - very much	Feeling relaxed	[9]
	Op dit moment voel ik me gewaardeerd	Right now, I feel appreciated	Helemaal niet - heel erg	Not at all - very much	Feeling appreciated	[9]
	Sinds het vorige meetmoment heb ik plezier gehad	Since the previous measurement, I've experienced pleasure	Helemaal niet - heel erg	Not at all - very much	Experiencing pleasure	[9]
	Ik heb zin in de rest van de dag	I look forward to the rest of the day	Helemaal niet - heel erg	Not at all - very much	Anticipatory reward	[2, 10]
			**Main construct: Formulate your own questions**			
**Subconstruct**	**EMA item (NL)**	**EMA item (EN)**	**Response scale (NL)**	**Response scale (EN)**	**Construct**	**Reference**

**Formulate your own questions**	- Op dit moment [typ hier je eigen vraag]<br>- Sinds het vorige meetmoment [typ hier je eigen vraag	- Right now [enter your question here]<br>- Since the previous measurement [enter your question here]	Helemaal niet - heel erg	Not at all - very much	Person-specific item	[21]
	- Op dit moment [typ hier je eigen vraag]<br>- Sinds het vorige meetmoment [typ hier je eigen vraag	- Right now [enter your question here]<br>- Since the previous measurement [enter your question here]	Helemaal niet - heel erg	Not at all - very much	Person-specific item	[21]
	- Op dit moment [typ hier je eigen vraag]<br>- Sinds het vorige meetmoment [typ hier je eigen vraag	- Right now [enter your question here]<br>- Since the previous measurement [enter your question here]	Helemaal niet - heel erg	Not at all - very much	Person-specific item	[21]
	- Op dit moment [typ hier je eigen vraag]<br>- Vandaag [typ hier je eigen vraag]	- Right now [enter your question here]<br>- Today [enter your question here]	Helemaal niet - heel erg	Not at all - very much	Person-specific item (evening)	

Fixed beep schedule 3, 5, 7, or 10 times per day

Constructs, subconstructs, and EMA items

Main construct: Mood						
**Subconstruct**	**EMA item (NL)**	**EMA item (EN)**	**Response scale (NL)**	**Response scale (EN)**	**Construct**	**Reference**
**Mood (obligatory)**	Op dit moment voel ik me opgewekt	Right now, I feel cheerful	Helemaal niet - heel erg	Not at all - very much	PA high arousal	[2, 8-12]
	Op dit moment voel ik me somber	Right now, I feel down	Helemaal niet - heel erg	Not at all - very much	NA low arousal	[8-13]
	Op dit moment voel ik me tevreden	Right now, I feel content	Helemaal niet - heel erg	Not at all - very much	PA low arousal	[2, 8-12]
	Op dit moment voel ik me geïrriteerd	Right now, I feel irritated	Helemaal niet - heel erg	Not at all - very much	NA high arousal	[2, 10-13]
	Op dit moment voel ik me energiek	Right now, I feel energetic	Helemaal niet - heel erg	Not at all - very much	PA high arousal	[8-12]
	Op dit moment voel ik me onrustig	Right now, I feel restless	Helemaal niet - heel erg	Not at all - very much	NA high arousal	[8-11]
**Standard last item of the EMA question-naire**	Als u nog iets anders over de periode sinds het vorige meetmoment wilt noteren, dan kunt u dat hier doen.	If you would like to note down something else about the period since the previous measurement, you can do that here.	[String (optioneel), max 280 karakters]	[String (optional), max 280 characters]	Qualitative description of anything of relevance not captured by the other items	
**Main construct: (Hypo)mania**						
**Subconstruct**	**EMA item (NL)**	**EMA item (EN)**	**Response scale (NL)**	**Response scale (EN)**	**Construct**	**Reference**
**(Hypo)mania**	Op dit moment voel ik me bijzonder goed	Right now, I feel extremely well	Helemaal niet - heel erg	Not at all - very much	Expansive mood	[2]
	Op dit moment voel ik me gejaagd	Right now, I feel agitated	Helemaal niet - heel erg	Not at all - very much	Agitation	[2, 14]
	Op dit moment voel ik me prikkelbaar	Right now, I feel irritable	Helemaal niet - heel erg	Not at all - very much	Irritable mood	[9]

	Op dit moment gaan mijn gedachten snel	Right now, my thoughts are racing	Helemaal niet - heel erg	Not at all - very much	Racing thoughts	[2, 14]
	Op dit moment ben ik snel afgeleid	Right now, I am easily distracted	Helemaal niet - heel erg	Not at all - very much	Distractability	[2, 9]
	Op dit moment zit ik vol goede ideeën	Right now, I am full of good ideas	Helemaal niet - heel erg	Not at all - very much	Expansive ideas	[2, 15]
	Op dit moment heb ik zin om met anderen af te spreken	Right now, I feel like socializing	Helemaal niet - heel erg	Not at all - very much	Need for social contact	[2]
	Op dit moment kan ik snel schakelen	Right now, I can focus and switch easily	Helemaal niet - heel erg	Not at all - very much	Thinking clearly	[2, 14]
	Sinds het vorige meetmoment heb ik contact gezocht met anderen	Since the previous measurement, I have sought contact with others	Helemaal niet - heel erg	Not at all - very much	Actual social contact	[2]
	Sinds het vorige meetmoment ben ik spraakzaam geweest	Since the previous measurement, I have been talkative	Helemaal niet - heel erg	Not at all - very much	Talkativeness	[2, 14]
			**Main construct: Agitation and stress**			
**Subconstruct**	**EMA item (NL)**	**EMA item (EN)**	**Response scale (NL)**	**Response scale (EN)**	**Construct**	**Reference**
**Agitation**	Op dit moment voel ik me gestrest	Right now, I feel stressed	Helemaal niet - heel erg	Not at all - very much	Stressed feeling	[10]
	Op dit moment voel ik me wantrouwig	Right now, I feel suspicious	Helemaal niet - heel erg	Not at all - very much	Paranoid delusion	[16, 17]
	Op dit moment voel ik me gespannen	Right now, I feel tense	Helemaal niet - heel erg	Not at all - very much	Stressed feeling	[10]
	Op dit moment voel ik me gejaagd	Right now, I feel agitated	Helemaal niet - heel erg	Not at all - very much	Agitation	[2, 14]
	Op dit moment voel ik me prikkelbaar	Right now, I feel irritable	Helemaal niet - heel erg	Not at all - very much	Irritable mood	[9]
**Over-stimulation**	Op dit moment voel ik me overprikkeld	Right now, I feel over-stimulated	Helemaal niet - heel erg	Not at all - very much	Over-stimulation	Adapted from[18]

	Op dit moment is het een chaos in mijn hoofd	Right now, my mind is reeling	Helemaal niet - heel erg	Not at all - very much	Disorganized thinking	[17]
	Op dit moment ben ik snel afgeleid	Right now, I am easily distracted	Helemaal niet - heel erg	Not at all - very much	Concentration/ distractability	[9]
	Op dit moment kan ik me goed concentreren	Right now, I can concentrate well	Helemaal niet - heel erg	Not at all - very much	Concentration	[10, 12, 17]
**Stress**	Op dit moment voel ik me gestrest	Right now, I feel stressed	Helemaal niet - heel erg	Not at all - very much	Stressed feeling	[19]
	Op dit moment zie ik op tegen de rest van de dag	Right now, I am dreading the rest of the day	Helemaal niet - heel erg	Not at all - very much		[10]
	Denk aan de meest plezierige gebeurtenis of activiteit sinds het vorige meetmoment. Hoe plezierig was deze gebeurtenis?	Think of the most pleasant event or activity since the previous measurement. How pleasurable was this event?	Helemaal niet - heel erg plezierig	Not pleasant at all - very pleasant	Reward sensitivity	[11, 12, 19]
	Hoe heftig was deze gebeurtenis?	How intense was this event?	Helemaal niet - heel erg	Not at all - very much	Reward sensitivity	[11]
	De plezierige ervaring had te maken met:	The pleasant event concerned:	[String (optioneel), max 280 karakters]	[String (optional), max 280 characters]	Qualitative description of event	[11]
	Denk aan de meest onplezierige gebeurtenis of activiteit sinds het vorige meetmoment. Hoe onplezierig was deze gebeurtenis?	Think of the most unpleasant event or activity since the previous measurement. How unpleasant was this event?	Helemaal niet onplezierig - heel erg onplezierig	Not unpleasant at all - very unpleasant	Stress reactivity	[11, 12, 19]
	Hoe heftig was deze gebeurtenis?	How intense was this event?	Helemaal niet - heel erg	Not at all - very much	Stress reactivity	[11]

	De onplezierige ervaring had te maken met	The unpleasant event concerned:	[String (optioneel), max 280 karakters]	[String (optional), max 280 characters]	Qualitative description of event	[11]
			**Main construct: Anxiety**			
**Subconstruct**	**EMA item (NL)**	**EMA item (EN)**	**Response scale (NL)**	**Response scale (EN)**	**Construct**	**Reference**
	Op dit moment voel ik me veilig	Right now, I feel safe	Helemaal niet - heel erg	Not at all - very much	Feeling safe	[20]
	Op dit moment voel ik me gespannen	Right now, I feel tense	Helemaal niet - heel erg	Not at all - very much	Stressed feeling	[19]
	Sinds het vorige meetmoment heb ik gepiekerd	Since the previous measurement, I have worried	Helemaal niet - heel erg	Not at all - very much	Worrying	[10, 11]
	Sinds het vorige meetmoment heb ik iets afgezegd of vermeden	Since the previous measurement, I have cancelled or avoided something	Helemaal niet - heel erg	Not at all - very much	Avoidance	[21]
	Op dit moment ben ik bang om alleen te zijn	Right now, I am scared of being alone	Helemaal niet - heel erg	Not at all - very much	Anxiety for being alone	[21]
**Intrusions and compulsive complaints**	Sinds het vorige meetmoment had ik last van nare herinneringen	Since the previous measurement, I had intrusive memories	Helemaal niet - heel erg	Not at all - very much	Intrusions	[21]
	Sinds het vorige meetmoment heb ik dwangklachten gehad	Since the previous measurement, I had compulsive complaints	Helemaal niet - heel erg	Not at all - very much	Compulsive complaints	[21]
**Panic**	Op dit moment ben ik bang dat ik een paniekaanval krijg	Right now, I am scared of having a panic attach	Helemaal niet - heel erg	Not at all - very much	Anxiety for panic attacks	[21]
	Sinds het vorige meetmoment had ik last van paniekklachten	Since the previous measurement, I had panic complaints (f.e.,	Helemaal niet - heel erg	Not at all - very much	Physical anxiety complaints	[21]

	(bijv. hartkloppingen, zweten, misselijkheid)	heart palpitations, sweating, nausea)				
**Derealization**	Op dit moment voel ik me onwerkelijk	Right now, I feel surreal	Helemaal niet - heel erg	Not at all - very much	Derealization	[21]
	Op dit moment ben ik bang om de controle te verliezen	Right now, I am scared of losing control	Helemaal niet - heel erg	Not at all - very much	Anxiety for losing control	[21]
			**Main construct: Psychosis**			
**Subconstruct**	**EMA item (NL)**	**EMA item (EN)**	**Response scale (NL)**	**Response scale (EN)**	**Construct**	**Reference**
**Hearing voices**	Sinds het vorige meetmoment hoorde ik stemmen	Since the previous measurement, I heard voices	- Ja<br>- Nee	- Yes<br>- No	Hearing voices	[20]
(if yes on first item)	Sinds het vorige meetmoment had ik last van de stemmen	Since the previous measurement, I was bothered by the voices	Helemaal niet - heel erg	Not at all - very much	Bothered by the voices	Adapted from [22]
(if yes on first item)	Wat was de voornaamste copingstrategie die je hebt gebruikt tegen de stemmenlast?	What was your main coping strategy that you used against the burden you experienced by the voices?	**Checklist: (één optie mogelijk)**<br>- Doen wat de stemmen zeggen<br>- In gesprek gaan met de stemmen<br>- De stemmen wegsturen of zeggen te stoppen<br>- Afleiding door iets sociaals<br>- Afleiding door iets taligs (bijv. lezen, zingen)<br>- Afleiding door werkgeheugenbelasting (bijv. TemStem, woordpuzzel)<br>- Blowen of alcohol drinken	**Checklist (one option possible)**<br>- Do what voices say<br>- Talk to voices<br>- Send voices away or order to stop<br>- Distract by doing something social<br>- Distract by something verbal (f.e., reading, singing)<br>- Distract by taxing the working memory (f.e., TemStem, word puzzle)<br>- Using drugs or alcohol<br>- Taking medication	Coping with the voices	Adapted from [22]

			- Medicatie innemen<br>- Iets ontspannens (bijv. sporten, muziek luisteren)<br>- Geruststellende gedachten<br>- Ik gebruikte geen copingstrategie<br>- Iets anders	- Something relaxing (f.e., doing sports, listening to music)<br>- Reassuring thoughts<br>- I did not use a coping strategy<br>- Something else		
(if yes on first item)	In welke mate heeft deze copingstrategie je geholpen de stemmenlast te verminderen?	To what extent has the coping strategy helped you to reduce the burden of the voices?	Helemaal niet - heel erg	Not at all - very much	Effectivity of coping strategies	Adapted from [22]
(if yes on first item)	De stemmen maakten mij boos	The voices made me angry	Helemaal niet - heel erg	Not at all - very much	Emotions due to the voices	Adapted from [22]
(if yes on first item)	De stemmen maakten mij blij	The voices made me happy	Helemaal niet - heel erg	Not at all - very much	Emotions due to the voices	Adapted from [22]
(if yes on first item)	De stemmen maakten mij bedroefd	The voices made me sad	Helemaal niet - heel erg	Not at all - very much	Emotions due to the voices	Adapted from [22]
(if yes on first item)	De stemmen maakten mij beschaamd	The voices made me feel ashamed	Helemaal niet - heel erg	Not at all - very much	Emotions due to the voices	Adapted from [22]
(if yes on first item)	De stemmen maakten mij bang	The voices scared me	Helemaal niet - heel erg	Not at all - very much	Emotions due to the voices	Adapted from [22]
**Delusions**	Op dit moment voel ik me wantrouwig	Right now, I feel suspicious	Helemaal niet - heel erg	Not at all - very much	Paranoid delusion	[13, 17, 20]
	Op dit moment voel ik me veilig	Right now, I feel safe	Helemaal niet - heel erg	Not at all - very much	Paranoid delusion	[20]
	Op dit moment lijkt alles betekenis te hebben	Right now, I feel as if	Helemaal niet - heel erg	Not at all - very much	Verhoogde betekenisgeving	[13]
	Op dit moment voel ik me onwerkelijk	Right now, I feel surreal	Helemaal niet - heel erg	Not at all - very much	Derealization	[21]

	Het is weer bezig	It is going on again	Helemaal niet - heel erg	Not at all - very much	Person-specific delusion	[23]
	Op dit moment worden mijn gedachten door anderen beïnvloed	Right now, my thoughts are influenced by others	Helemaal niet - heel erg	Not at all - very much	Beïnvloedingswaan	[13, 17, 20]
			**Main construct: Bodily complaints**			
**Subconstruct**	**EMA item (NL)**	**EMA item (EN)**	**Response scale (NL)**	**Response scale (EN)**	**Construct**	**Reference**
**Bodily complaints**	Sinds het vorige meetmoment heb ik last gehad van:	Since the previous measurement, I have been bothered by:	**Checklist (meerdere opties mogelijk):**<br>- Ademhalingsproblemen (kortademigheid, moeilijk adem kunnen krijgen)<br>- Pijn of drukkend gevoel op de borst<br>- Hartkloppingen<br>- Misselijkheid of maag die van streek is<br>- Verstopping, dunne ontlasting of diarree<br>- Pijn in de buik of maagstreek<br>- Hoofdpijn<br>- Duizeligheid<br>- Vermoeidheid<br>- Rugpijn<br>- Pijn in spieren of gewrichten<br>- Verdoofd of tintelend gevoel	**Checklist (multiple responses possible):**<br>- Trouble breathing (shortness of breath, breathing difficulties)<br>- Pain or pressing feeling on the chest<br>- Heart palpitations<br>- Nausea or upset stomach<br>- Constipation, diarrhea<br>- Abdominal pain or stomachaches<br>- Headache<br>- Dizziness<br>- Fatigue<br>- Back pain<br>- Pain in joints or muscles<br>- Numbness or tingling	Bodily complaints	Adapted from [24]
(for each selected complaint)	Hoeveel had je last van [klacht]	How much were you bothered by [complaint]	Helemaal niet - heel erg	Not at all - very much	Bodily complaints	[25]

Subconstruct	EMA item (NL)	EMA item (EN)	Response scale (NL)	Response scale (EN)	Construct	Reference
	Sinds het vorige meetmoment was ik in gedachten veel met mijn lichamelijke klachten bezig	Since the previous measurement, I was preoccupied by my bodily complaints	Helemaal niet - heel erg	Not at all - very much	Preoccupation with bodily complaints	[25]
	Sinds het vorige meetmoment zocht ik afleiding van mijn lichamelijke klachten	Since the previous measurement, I sought distraction from my bodily complaints	Helemaal niet - heel erg	Not at all - very much	Seeking distraction from bodily complaints	[25]
	Sinds het vorige meetmoment voelde ik mij belemmerd door mijn lichamelijke klachten	Since the previous measurement, I felt hindered by my bodily complaints	Helemaal niet - heel erg	Not at all - very much	Feeling hindered by bodily complaints	[25]
			**Main construct: Reduced interest**			
**Subconstruct**	**EMA item (NL)**	**EMA item (EN)**	**Response scale (NL)**	**Response scale (EN)**	**Construct**	**Reference**
**Reduced interest and initiative**	Ik heb zin in de rest van de dag	I look forward to the rest of the day	Helemaal niet - heel erg	Not at all - very much	Anticipatory reward	[2]
	Op dit moment heb ik zin om met anderen te zijn	Right now, I feel like being with others	Helemaal niet - heel erg	Not at all - very much	Need for socializing	[10]
	Op dit moment voel ik me lusteloos	Right now, I feel listless	Helemaal niet - heel erg	Not at all - very much	Anhedonia	[10, 12]
			**Main construct: Surroundings, behavior, agents**			
**Subconstruct**	**EMA item (NL)**	**EMA item (EN)**	**Response scale (NL)**	**Response scale (EN)**	**Construct**	**Reference**
**Events**	Denk aan de meest plezierige gebeurtenis of activiteit sinds het vorige meetmoment. Hoe plezierig was dit?	Think of the most pleasant event or activity since the previous measurement. How pleasurable was this?	Helemaal niet - heel erg plezierig	Not at all - very pleasant	Reward sensitivity	[11, 19]

	Hoe heftig was deze gebeurtenis of activiteit?	How intense was this event?	Helemaal niet - heel erg	Not at all - very much	Reward sensitivity	[11]
	De plezierige ervaring had te maken met:	The pleasant event concerned:	[String (optioneel), max 280 karakters]	[String (optional), max 280 characters]	Qualitative description of event	[11, 19]
	Denk aan de meest onplezierige gebeurtenis of activiteit sinds het vorige meetmoment. Hoe onplezierig was dit?	Think of the most unpleasant event or activity since the previous measurement. How unpleasant was this?	Helemaal niet - heel erg onplezierig	Not at all – very unpleasant	Stress reactivity	[11]
	Hoe heftig was dit?	How intense was this?	Helemaal niet - heel erg	Not at all - very much	Stress reactivity	[11]
	De onplezierige gebeurtenis had te maken met	The unpleasant event concerned:	[String (optioneel), max 280 karakters]	[String (optional), max 280 characters]	Qualitative description of event	[11]
**Activity**	Op dit moment, wat doe ik?	Right now, what am I doing?	**Checklist (één optie mogelijk):**<br>- Werken/studeren<br>- Sporten/wandelen/ fietsen<br>- Hobby (bijv. muziek maken, klussen)<br>- Uitje (bijv. naar de stad, concert)<br>- Iets rustigs (bijv. lezen, tv kijken)<br>- Met iemand praten<br>- Iets intiems (bijv. knuffelen, seks)<br>- Whatsappen	**Checklist (one option possible):**<br>- Working/studying<br>- Doing sports/walking/ biking<br>- Hobby (f.e., making music, DIY)<br>- Outing (f.e., going into the city or a concert)<br>- Something relaxing (f.e., reading, watching TV)<br>- Talking to someone<br>- Something intimate (cuddling, sex)<br>- Whatsapp/texting	(social) context	[9-11]

			- Social Media<br>- Eten<br>- Huishouden/ boodschappen/ administratie<br>- Zelfverzorging (bijv. douchen, opmaken)<br>- Rusten/niets<br>- Ik ben onderweg<br>- Iets anders	- Social Media<br>- Eating<br>- Household chores/ grocery shopping/ administration<br>- Self-care (f.e., showering, applying make-up)<br>- Resting/nothing<br>- Travelling, or moving from one place to another<br>- Something else		
	Sinds het vorige meetmoment, wat heb ik gedaan?	Since the previous measurement, what have I been doing?	**Checklist (meerdere opties mogelijk):**<br>[Hetzelfde als bovenstaand item]	**Checklist (multiple responses possible):**<br>[same as previous item]		[9-11]
	Deze activiteit vind ik…	I find this activity…	Zeer onplezierig - zeer plezierig	Very unpleasant – very pleasant		[9-11]
	Sinds het vorige meetmoment ben ik lichamelijk actief geweest	Since the previous measurement, I have been physically active	Helemaal niet - heel erg	Not at all - very much	Physical activity	[10, 11, 26]
**Surroundings**	Sinds het vorige meetmoment ben ik buiten geweest	Since the previous measurement, I have been outside	- Ja<br>- Nee	- Yes<br>- No	Being outside	[21]
	Op dit moment, waar ben ik?	Right now, where am I?	**Checklist (één optie mogelijk):**<br>- Thuis<br>- School/werk<br>- Bij familie/vrienden<br>- Café, bibliotheek, park<br>- Supermarkt/winkel	**Checklist (one option possible):**<br>- Home<br>- School/work<br>- Family/friends<br>- Cafe/library/park<br>- Supermarket/shop	Location	[9]

			- In de natuur<br>- Hulpverlening<br>- Onderweg<br>- Ergens anders	- Nature<br>- Health facility<br>- Travelling, or moving from one place to another<br>- Somewhere else		
**Food, agents, and side-effects**	Sinds het vorige meetmoment heb ik de volgende middelen gebruikt	Since the previous measurement, I have taken the following	**Checklist (meerdere opties mogelijk):**<br>- Geen<br>- Sigaretten<br>- Koffie of energydrank<br>- Alcohol<br>- Snacks<br>- Snoep<br>- Cannabis<br>- Medicatie	**Checklist (multiple responses possible):**<br>- None<br>- Cigarettes<br>- Coffee or energy drink<br>- Alcohol<br>- Snacks<br>- Candy<br>- Cannabis<br>- Medication	Middelen/medicatiegebruik	[17]
	Sinds het vorige meetmoment heb ik deze ervaring(en) gehad	Since the previous measurement, I have had the following experiences	**Checklist (meerdere opties mogelijk):**<br>- Hoofdpijn<br>- Hongergevoel<br>- Misselijkheid<br>- Suf of slaperig<br>- Pijnlijke of stijve spieren<br>- Onrust van binnen<br>- Duizelig<br>- Droge mond<br>- Vlak<br>- Traag<br>- Geen van bovenstaande	**Checklist (multiple responses possible):**<br>- Headache<br>- Hunger<br>- Nausea<br>- Drowsy or sleepy<br>- Painful or stiff muscles<br>- Restlessness<br>- Dizziness<br>- Dry mouth<br>- Flat<br>- Slow<br>- None of the above	Medication side-effects	[21]
**Food**	Sinds het vorige meetmoment heb ik het volgende gegeten	Since the previous measurement, I have eaten the following	**Checklist (meerdere opties mogelijk):**<br>- Broodmaaltijd	**Checklist (multiple responses possible):**<br>- Sandwich	Food type	[21]

			- Gezond tussendoortje<br>- Warme maaltijd<br>- Snoep<br>- Snacks<br>- Niets	- Healthy snack<br>- Hot meal<br>- Candy<br>- Snack<br>- Nothing		
	Sinds het vorige meetmoment heb ik gegeten…	Since the previous measurement, I have eaten…	Veel minder dan normaal - veel meer dan normaal	A lot less than usual – a lot more than usual	Food quantity	[21]
	Sinds het vorige meetmoment heb ik gezond gegeten	Since the previous measurement, I have eaten healthily	Helemaal niet - heel erg	Not at all - very much	Health level of food	[21]
			**Hoofdthema: Social interactions**			
**Subconstruct**	**EMA item (NL)**	**EMA item (EN)**	**Response scale (NL)**	**Response scale (EN)**	**Construct**	**Reference**
**Company**	Op dit moment ben ik…	Right now, I am…	**Checklist (één optie mogelijk):**<br>- Alleen<br>- Alleen met huisdier<br>- In gezelschap	**Checklist (one response possible):**<br>- Alone<br>- Alone with pet<br>- In company	Alone/with others	[11]
(if in company)	Op dit moment, met wie ben ik?	Right now, with whom am I?	**Checklist (meerdere opties mogelijk):**<br>- Partner<br>- Huisgenoten<br>- Gezin<br>- Familie uitwonend<br>- Vrienden<br>- Collega's/ klasgenoten<br>- Hulpverleners<br>- Kennissen<br>- Onbekenden	**Checklist (multiple responses possible):**<br>- Partner<br>- House mates<br>- Family<br>- Extended family<br>- Friends<br>- Colleagues/class mates<br>- Clinicians<br>- Acquaintances<br>- Strangers	Company type	[9, 11, 13]
(if in company)	Vul hier in met wie je bent. *NB: noteer hier geen namen en*	Indicate with whom you are. *Note: do not mention names here. Remember privacy.*	[String (optioneel), max 280 karakters]	[String (optional), max 280 characters]		[27-29]

	dergelijke. Denk om privacy.					
(if in company)	Ik vind dit gezelschap aangenaam	I find this company agreeable	Helemaal niet - heel erg	Not at all - very much	Social stress	[9, 11, 30]
(if in company)	Ik heb het gevoel dat deze mensen me mogen	I feel these people like me	Helemaal niet - heel erg	Not at all - very much	Social stress	[30]
(if alone)	Ik was liever in gezelschap geweest	I'd rather have been in company	Helemaal niet - heel erg	Not at all - very much	Social stress	[13, 30]
(if alone)	Ik vind het fijn om nu alleen te zijn	Right now, I like being alone	Helemaal niet - heel erg	Not at all - very much	Social stress	[30]
(if in company)	Ik voel me geaccepteerd in dit gezelschap	I feel accepted by this company	Helemaal niet - heel erg	Not at all - very much	Social stress	[11, 30]
(if in company)	Ik voel me op mijn gemak in dit gezelschap	I feel at ease in this company	Helemaal niet - heel erg	Not at all - very much	Social stress	[30]
**Connected-ness**	Sinds het vorige meetmoment voelde ik me eenzaam	Since the previous measurement, I have felt lonely	Helemaal niet - heel erg	Not at all - very much	Loneliness	[9, 13]
	Op dit moment voel ik dat ik om anderen geef	Right now, I feel I care for others	Helemaal niet - heel erg	Not at all - very much	Caring for others	[27-29]
	Op dit moment voel ik dat anderen om mij geven	Right now, I feel that others care about me	Helemaal niet - heel erg	Not at all - very much	Feeling others care about you	[17, 27-29]
	Op dit moment voel ik me verbonden met anderen	Right now, I feel connected with others	Helemaal niet - heel erg	Not at all - very much	Feeling connected	[27-29]
**Interactions**	Heb je sinds het vorige meetmoment gesprekken gehad? *Gesprekken duren langer dan 5 minuten. Dit kunnen face-to-face*	Since the previous measurement, did you have interactions? *Interactions last longer than 5 minutes, and can be face-to-face*	- Ja<br>- Nee	- Yes<br>- No	Had interactions	[27-29]

	gesprekken of gesprekken via online (sociale) media zijn.	*interactions or via online (social) media.*				
	Sinds het vorige meetmoment, hoeveel gesprekken heb je gehad?	Since the previous measurement, how many interactions did you have?	[Geef getal aan]	[indicate number]	Number of interactions	[27-29]
	Denk aan het voor jou meest belangrijk gesprek sinds het vorige meetmoment. Met wie heb je dit gesprek gehad? *NB: noteer hier geen namen en dergelijke. Denk om privacy.*	Think about the most important interaction since the previous measurement. With whom did you have this interaction? *Note: do not mention names here. Remember privacy.*	[String (optioneel), max 280 karakters]	[String (optional), max 280 characters]	Interaction partner	[27-29]
	Wat voor gesprek was dit?	What kind of interaction was this?	**Checklist (één optie mogelijk):**<br>- Face-to-face<br>- Telefoongesprek<br>- Videobelgesprek<br>- Gesprek via email of sociale media (bijv. Whatsapp)<br>- Iets anders	**Checklist (one option possible):**<br>- Face-to-face<br>- By phone<br>- By video messaging<br>- By e-mail or social media (f.e., whatsapp)<br>- Something else	Medium	[27-29]
	Hoe belangrijk was dit gesprek voor je?	How important was this interaction for you?	Helemaal niet - heel erg	Not at all - very much	Interaction importance	[27-29]
	Wat maakte dit gesprek belangrijk en waar ging het over?	What made this interaction important and what was it about?	[String (optioneel), max 280 karakters]	[String (optional), max 280 characters]	Qualitative description of interaction importance	[27-29]

	Hoe lang duurde het gesprek?	What was the duration of the interaction?	[Geef getal aan (minuten)]	[Indicate number (minutes)]	Interaction duration	[27-29]
	Hoe (on)plezierig was de inhoud van het gesprek?	How (un)pleasant was the content of the interaction?	Heel erg onplezierig – heel erg plezierig	Very unpleasant – very pleasant	(Un)pleasantnes s of interaction content	[27-29]
	Hoe (on)plezierig heb je het gesprek zelf ervaren? *Denk hierbij bijvoorbeeld aan de houding en toon van je gesprekspartner.*	How (un)pleasant was the interaction itself? *Think about the attitude and tone of your interaction partner.*	Heel erg onplezierig – heel erg plezierig	Very unpleasant – very pleasant	(Un)pleasant-ness of interaction itself	[27-29]
	In hoeverre beïnvloedde het gesprek je stemming?	To what extent did the interaction influence your mood?	Mijn stemming werd veel slechter - mijn stemming werd veel beter	My mood worsened a lot – my mood improved a lot	Impact of interaction on mood	[27-29]
	In hoeverre beïnvloedde het gesprek je energieniveau?	To what extent did the interaction influence your energy level?	Het kostte me veel energie - Het leverde me veel energie op	It cost me a lot of energy – it gave me a lot of energy	Impact of interaction on energy level	[27-29]
	In hoeverre kon je jezelf zijn in dit gesprek?	To what extent could you be yourself in this interaction?	Helemaal niet - heel erg	Not at all - very much	Being yourself	[27-29]
	Ik voelde me gesteund door mijn gesprekspartner(s)	I felt supported by my interaction partner(s).	Helemaal niet - heel erg	Not at all - very much	Feeling supported	[27-29]
	Is er sinds het vorige meetmoment nog een ander gesprek geweest dat je graag wilt beschrijven?	Has there been another interaction since the previous measurement that you would like to describe?	- Ja<br>- Nee<br>[indien ja, herhaal vorige items]	- Yes<br>- No<br>[if yes, repeat previous items]	Another interaction	[27-29]
			**Main construct: Worrying**			
**Subconstruct**	**EMA item (NL)**	**EMA item (EN)**	**Response scale (NL)**	**Response scale (EN)**	**Construct**	**Reference**

**Worrying**	Sinds het vorige meetmoment heb ik gepiekerd	Since the previous measurement, I have worried	Helemaal niet - heel erg	Not at all - very much	Worrying	[10, 11]
	Op dit moment laten mijn gedachten me niet los	Right now, I can't disconnect from my thoughts	Helemaal niet - heel erg	Not at all - very much	Worrying	[17]
			**Main construct: Self-esteem**			
**Subconstruct**	**EMA item (NL)**	**EMA item (EN)**	**Response scale (NL)**	**Response scale (EN)**	**Construct**	**Reference**
**Self-esteem**	Op dit moment voel ik me goed over mezelf	Right now, I feel good about myself	Helemaal niet - heel erg	Not at all - very much	Self-esteem	[17]
	Op dit moment zijn mijn gedachten over mezelf…	Right now, my thoughts about myself are…	Heel erg negatief – heel erg positief	Very negative – very positive	Self-esteem	[10]
	Op dit moment heb ik het gevoel te kort te schieten	Right now, I feel inadequate	Helemaal niet - heel erg	Not at all - very much	Self-esteem	[9, 10]
	Op dit moment voel ik me nuttig	Right now, I feel useful	Helemaal niet - heel erg	Not at all - very much	Productivity	[2]
			**Main construct: Positivity**			
**Subconstruct**	**EMA item (NL)**	**EMA item (EN)**	**Response scale (NL)**	**Response scale (EN)**	**Construct**	**Reference**
**Positivity**	Op dit moment voel ik me goed over mezelf	Right now, I feel good about myself	Helemaal niet - heel erg	Not at all - very much	Self-esteem	[21]
	Op dit moment heb ik zin om met anderen te zijn	Right now, I feel like socializing	Helemaal niet - heel erg	Not at all - very much	Need for social contact	[10]
	Op dit moment voel ik me ontspannen	Right now, I feel relaxed	Helemaal niet - heel erg	Not at all - very much	Feeling relaxed	[9]
	Op dit moment voel ik me gewaardeerd	Right now, I feel appreciated	Helemaal niet - heel erg	Not at all - very much	Feeling appreciated	[9]
	Sinds het vorige meetmoment heb ik plezier gehad	Since the previous measurement, I've experienced pleasure	Helemaal niet - heel erg	Not at all - very much	Experiencing pleasure	[9]

	Ik heb zin in de rest van de dag	I look forward to the rest of the day	Helemaal niet - heel erg	Not at all - very much	Anticipatory reward	[2, 10]
			**Main construct: Formulate your own questions**			
**Subconstruct**	**EMA item (NL)**	**EMA item (EN)**	**Response scale (NL)**	**Response scale (EN)**	**Construct**	**Reference**
**Formulate your own questions**	- Op dit moment [typ hier je eigen vraag]<br>- Sinds het vorige meetmoment [typ hier je eigen vraag	- Right now [enter your question here]<br>- Since the previous measurement [enter your question here]	Helemaal niet - heel erg	Not at all - very much	Person-specific item	[21]
	- Op dit moment [typ hier je eigen vraag]<br>- Sinds het vorige meetmoment [typ hier je eigen vraag	- Right now [enter your question here]<br>- Since the previous measurement [enter your question here]	Helemaal niet - heel erg	Not at all - very much	Person-specific item	[21]
	- Op dit moment [typ hier je eigen vraag]<br>- Sinds het vorige meetmoment [typ hier je eigen vraag	- Right now [enter your question here]<br>- Since the previous measurement [enter your question here]	Helemaal niet - heel erg	Not at all - very much	Person-specific item	[21]
	- Op dit moment [typ hier je eigen vraag]<br>- Vandaag [typ hier je eigen vraag]	- Right now [enter your question here]<br>- Today [enter your question here]	Helemaal niet - heel erg	Not at all - very much	Person-specific item (evening)	[21]

Once-per-day evening schedule

Constructs, subconstructs, and EMA items

Subconstruct	EMA item (NL)	EMA item (EN)	Response scale (NL)	Response scale (EN)	Construct	Reference
			**Main construct: Mood**			
**Subconstruct**	**EMA item (NL)**	**EMA item (EN)**	**Response scale (NL)**	**Response scale (EN)**	**Construct**	**Reference**
**Mood (obligatory)**	Vandaag was mijn stemming…	Today, my mood was…	Heel erg slecht - heel erg goed	Very bad - very good	General mood	[18]
	Vandaag voelde ik me gestrest	Today, I felt stressed	Helemaal niet - heel erg	Not at all - very much	Stress	[18]
	Vandaag voelde ik me ontspannen	Today, I felt relaxed	Helemaal niet - heel erg	Not at all - very much	Relaxation	[18, 31]
	Vandaag kon ik genieten*	Today, I could enjoy things*	Helemaal niet - heel erg	Not at all - very much	Hedonia	[18]
	Vandaag vond ik mijn dag de moeite waard*	Today, I felt my day was worth living*	Helemaal niet - heel erg	Not at all - very much	Depression	[18, 31]
**Standard last item of the EMA question-naire**	Als u nog iets anders over de periode sinds het vorige meetmoment wilt noteren, dan kunt u dat hier doen.	If you would like to note down something else about the period since the previous measurement, you can do that here.	[String (optioneel), max 280 karakters]	[String (optional), max 280 characters]	Qualitative description of anything of relevance not captured by the other items	
			**Main construct: (Hypo)mania**			
**Subconstruct**	**EMA item (NL)**	**EMA item (EN)**	**Response scale (NL)**	**Response scale (EN)**	**Construct**	**Reference**
**(Hypo)mania**	Vandaag was mijn stemming…*	Today, my mood was…*	Depressief – manisch	Depressed – manic	LifeChart	Adapted from [32]
	Vandaag heb ik veel gekocht*	Today, I bought a lot*	Helemaal niet - heel erg	Not at all - very much	Buying urge	[21]
	Vandaag was ik de hele dag aan het praten*	Today, I was talking all day*	Helemaal niet - heel erg	Not at all - very much	Talking urge	[14]
	Vandaag deed ik dingen in een opwelling*	Today, I was impulsive*	Helemaal niet - heel erg	Not at all - very much	Impulsisvity	[18]
			**Main construct: Agitation and stress**			

Subconstruct	EMA item (NL)	EMA item (EN)	Response scale (NL)	Response scale (EN)	Construct	Reference
**Agitation**	Vandaag voelde ik me onrustig	Today, I felt restless	Helemaal niet - heel erg	Not at all - very much	Restlessness	[18, 31]
	Vandaag was ik geïrriteerd	Today, I felt irritated	Helemaal niet - heel erg	Not at all - very much	Externalizing behavior	[18, 31]
	Vandaag was ik opstandig	Today, I felt rebellious	Helemaal niet - heel erg	Not at all - very much	Externalizing behavior	[18]
	Vandaag was ik ongeduldig*	Today, I felt impatient*	Helemaal niet - heel erg	Not at all - very much	Externalizing behavior	[18]
	Vandaag was ik dwars*	Today, I was recalcitrant*	Helemaal niet - heel erg	Not at all - very much	Externalizing behavior	[18]
	Vandaag deed ik dingen in een opwelling*	Today, I was impulsive*	Helemaal niet - heel erg	Not at all - very much	Impulsivity	[18]
	Vandaag verveelde ik me snel*	Today, I was bored quickly*	Helemaal niet - heel erg	Not at all - very much	Externalizing behavior	[18]
**Over-stimulation**	Vandaag kwam alles harder binnen*	Today, I felt overstimulated*	Helemaal niet - heel erg	Not at all - very much	Over-stimulation	[18]
	Vandaag had ik het gevoel de controle te verliezen*	Today, I felt I was losing control*	Helemaal niet - heel erg	Not at all - very much	Losing control	[18]
	Vandaag raakte ik gauw van slag*	Today, I was easily upset*	Helemaal niet - heel erg	Not at all - very much	Rigidity	[18]
	Vandaag schrok ik snel*	Today, I was easily startled*	Helemaal niet - heel erg	Not at all - very much	Increased startle response/hyper focus	[18]
**Stress**	Vandaag raakte ik gauw van slag	Today, I was easily upset	Helemaal niet - heel erg	Not at all - very much	Rigidity	[18]
	Vandaag schrok ik snel	Today, I was easily startled	Helemaal niet - heel erg	Not at all - very much	Increased startle response/hyper focus	[18]

Subconstruct	EMA item (NL)	EMA item (EN)	Response scale (NL)	Response scale (EN)	Construct	Reference
	Vandaag stoorden anderen zich aan mij	Today, others were annoyed by me	Helemaal niet - heel erg	Not at all - very much	Annoys others	[18]
			**Main construct: Anxiety**			
**Subconstruct**	**EMA item (NL)**	**EMA item (EN)**	**Response scale (NL)**	**Response scale (EN)**	**Construct**	**Reference**
**Anxiety**	Vandaag heb ik iets afgezegd of vermeden	Today, I have cancelled or avoided something	Helemaal niet - heel erg	Not at all - very much	Avoidance	[18]
	Vandaag heb ik contact opgenomen met veilige personen*	Today, I have contacted safe persons*	Helemaal niet - heel erg	Not at all - very much	Safety behavior	[21]
	Vandaag voelde ik me veilig	Today, I felt safe	Helemaal niet - heel erg	Not at all - very much	Feeling safe	[21]
	Vandaag voelde ik me vreemd	Today, I felt strange	Helemaal niet - heel erg	Not at all - very much	Dissociation/ derealization	[18]
			**Main construct: Psychosis**			
**Subconstruct**	**EMA item (NL)**	**EMA item (EN)**	**Response scale (NL)**	**Response scale (EN)**	**Construct**	**Reference**
**Delusions**	Op dit moment voel ik me veilig	Right now, I feel safe	Helemaal niet - heel erg	Not at all - very much	Paranoid delusion	[18]
	Op dit moment voel ik me wantrouwig	Right now, I feel suspicious	Helemaal niet - heel erg	Not at all - very much	Paranoid delusion	[18]
	Op dit moment voel ik me vreemd	Right now, I feel strange	Helemaal niet - heel erg	Not at all - very much	Dissociation/ derealization	[18]
			**Main construct: Reduced interest and initiative**			
**Subconstruct**	**EMA item (NL)**	**EMA item (EN)**	**Response scale (NL)**	**Response scale (EN)**	**Construct**	**Reference**
**Reduced interest and initiative**	Vandaag kon ik me nergens toe zetten*	Today, I could not bring myself to do anything*	Helemaal niet - heel erg	Not at all - very much	Initiative	[18]
	Ik heb zin in morgen	I look forward to tomorrow	Helemaal niet - heel erg	Not at all - very much	Reward anticipation	[18, 31]
	Ik zie op tegen iets wat binnenkort gebeurt (een bepaalde situatie, activiteit, ontmoeting)	I'm dreading something that will happen soon	Helemaal niet - heel erg	Not at all - very much	Stress anticipation	[18]

			Main construct: Bodily complaints			
**Subconstruct**	**EMA item (NL)**	**EMA item (EN)**	**Response scale (NL)**	**Response scale (EN)**	**Construct**	**Reference**
**Bodily complaints**	Vandaag heb ik last gehad van:	Today, I have been bothered by:	**Checklist (meerdere opties mogelijk):**<br>- Ademhalingsproblemen (kortademigheid, moeilijk adem kunnen krijgen)<br>- Pijn of drukkend gevoel op de borst<br>- Hartkloppingen<br>- Misselijkheid of maag die van streek is<br>- Verstopping, dunne ontlasting of diarree<br>- Pijn in de buik of maagstreek<br>- Hoofdpijn<br>- Duizeligheid<br>- Vermoeidheid<br>- Rugpijn<br>- Pijn in spieren of gewrichten<br>- Verdoofd of tintelend gevoel	**Checklist (multiple responses possible):**<br>- Trouble breathing (shortness of breath, breathing difficulties)<br>- Pain or pressing feeling on the chest<br>- Heart palpitations<br>- Nausea or upset stomach<br>- Constipation, diarrhea<br>- Abdominal pain or stomachaches<br>- Headache<br>- Dizziness<br>- Fatigue<br>- Back pain<br>- Pain in joints or muscles<br>- Numbness or tingling	Bodily complaints	Adapted from [24]
(for each selected complaint)	Hoeveel had je last van [klacht]	How much were you bothered by [complaint]	Helemaal niet - heel erg	Not at all - very much	Bodily complaints	[25]
	Vandaag was ik in gedachten veel met mijn lichamelijke klachten bezig	Today, I was preoccupied by my bodily complaints	Helemaal niet - heel erg	Not at all - very much	Preoccupation with bodily complaints	[25]

Subconstruct	EMA item (NL)	EMA item (EN)	Response scale (NL)	Response scale (EN)	Construct	Reference
	Vandaag kon ik mijn lichamelijke klachten goed accepteren	Today, I could accept my bodily complaints well	Helemaal niet - heel erg	Not at all - very much	Acceptance of bodily complaints	[25]
	Vandaag gaven mijn lichamelijke klachten mij een gevoel van falen*	Today, my bodily complaints made me feel like a failure*	Helemaal niet - heel erg	Not at all - very much	Feeling of failure due to bodily complaints	[25]
	Vandaag schaamde ik mij voor mijn lichamelijke klachten*	Today, I felt ashamed for my bodily complaints*	Helemaal niet - heel erg	Not at all - very much	Feeling ashamed of bodily complaints	[25]
			**Main construct: Events**			
**Subconstruct**	**EMA item (NL)**	**EMA item (EN)**	**Response scale (NL)**	**Response scale (EN)**	**Construct**	**Reference**
**Events**	Vandaag heb ik leuke dingen gedaan*	Today, I did fun things*	Helemaal niet – heel erg	Not at all – very much	Having fun	[21]
	Denk aan de meest plezierige gebeurtenis of activiteit sinds het vorige meetmoment. Hoe plezierig was dit?	Think of the most pleasant event or activity since the previous measurement. How pleasurable was this?	Helemaal niet - heel erg plezierig	Not at all - very pleasant	Reward sensitivity	Adapted from [11, 19]
	De plezierige ervaring had te maken met:	The pleasant event concerned:	[String (optioneel), max 280 karakters]	[String (optional), max 280 characters]	Qualitative description of event	[11, 19]
	Denk aan de meest onplezierige gebeurtenis of activiteit sinds het vorige meetmoment. Hoe onplezierig was dit?	Think of the most unpleasant event or activity since the previous measurement. How unpleasant was this?	Helemaal niet - heel erg onplezierig	Not at all – very unpleasant	Stress reactivity	Adapted from [11, 19]
	De onplezierige gebeurtenis had te maken met	The unpleasant event concerned:	[String (optioneel), max 280 karakters]	[String (optional), max 280 characters]	Qualitative description of event	[11]

Subconstruct	EMA item (NL)	EMA item (EN)	Response scale (NL)	Response scale (EN)	Construct	Reference
			**Main construct: Worrying**			
**Subconstruct**	**EMA item (NL)**	**EMA item (EN)**	**Response scale (NL)**	**Response scale (EN)**	**Construct**	**Reference**
**Worrying**	Vandaag maakte ik me zorgen	Today, I have worried	Helemaal niet - heel erg	Not at all - very much	Worrying	[18]
	Vandaag was ik er met mijn hoofd niet bij*	Today, I was absentminded*	Helemaal niet - heel erg	Not at all - very much	Absentminded	[18]
	Vandaag kon ik moeilijk beslissingen nemen*	Today, I had difficulty making decisions	Helemaal niet - heel erg	Not at all - very much	Indecisiveness	[18]
			**Main construct: Self-esteem**			
**Subconstruct**	**EMA item (NL)**	**EMA item (EN)**	**Response scale (NL)**	**Response scale (EN)**	**Construct**	**Reference**
**Zelfbeeld**	Vandaag had ik vertrouwen in mezelf	Today, I trusted in myself	Helemaal niet - heel erg	Not at all - very much	Self-esteem	[18]
	Vandaag was ik bang om dingen fout te doen*	Today, I was afraid of making mistakes*	Helemaal niet - heel erg	Not at all - very much	Perfectionism	[18]
	Vandaag had ik het gevoel dat anderen mij mochten*	Today, I felt others liked me*	Helemaal niet - heel erg	Not at all - very much	Self-esteem	[18]
			**Main construct: Functioning and resilience**			
**Subconstruct**	**EMA item (NL)**	**EMA item (EN)**	**Response scale (NL)**	**Response scale (EN)**	**Construct**	**Reference**
**Functioning**	Vandaag voelde ik mij belemmerd door mijn psychische klachten*	Today, I felt hindered by my psychological complaints*	Helemaal niet - heel erg	Not at all - very much	Feeling burdened	Adapted from [33]
	Vandaag kon ik aan wat er op mijn pad kwam*	Today, I could handle what was thrown my way*	Helemaal niet - heel erg	Not at all - very much	Resilience	[31]
	Vandaag ervoer ik grip op hoe het met me gaat*	Today, I felt in control of my well-being*	Helemaal niet - heel erg	Not at all - very much	Self-management	[11]
	Vandaag heb ik leuke dingen gedaan*	Today, I did fun things*	Helemaal niet - heel erg	Not at all - very much	Having fun	[21]

**Resilience**	Vandaag kon ik aan wat er op mijn pad kwam*	Today, I could handle what was thrown my way*	Helemaal niet - heel erg	Not at all - very much	Resilience	[31]
	Op dit moment accepteer ik me zoals ik ben*	Right now, I accept myself the way I am*	Helemaal niet - heel erg	Not at all - very much		[11]
**Main construct: Sleep during the day**						
**Subconstruct**	**EMA item (NL)**	**EMA item (EN)**	**Response scale (NL)**	**Response scale (EN)**	**Construct**	**Reference**
**Sleep during the day**	Hoe vaak heb ik vandaag dutjes gedaan?	Today, how often did I take a nap?	[Vul getal in]	[Enter number]	Sleep disturbance	[34]
	Hoe lang heb ik vandaag dutjes gedaan?	Today, how long did I take naps?	[Vul getal in (minuten)]	[Enter number (minutes)]	Sleep disturbance	[34]
**Main construct: Formulate your own questions**						
**Subconstruct**	**EMA item (NL)**	**EMA item (EN)**	**Response scale (NL)**	**Response scale (EN)**	**Construct**	**Reference**
**Formulate your own questions**	- Op dit moment [typ hier je eigen vraag]<br>- Vandaag [typ hier je eigen vraag]*	- Right now [enter your question here]<br>- Today [enter your question here]*	Helemaal niet - heel erg	Not at all - very much	Person-specific item	[21]
	- Op dit moment [typ hier je eigen vraag]<br>- Vandaag [typ hier je eigen vraag]	- Right now [enter your question here]<br>- Today [enter your question here]	Helemaal niet - heel erg	Not at all - very much	Person-specific item	[21]
	- Op dit moment [typ hier je eigen vraag]<br>- Vandaag [typ hier je eigen vraag]	- Right now [enter your question here]<br>- Today [enter your question here]	Helemaal niet - heel erg	Not at all - very much	Person-specific item	[21]

Note. The once-per-day schedules often are an insightful addition to the semi-random and fixed EMA schedules. Therefore, the PETRA decision aid has integrated some of the items of the once-per-day-schedules in the relevant (sub)constructs of the more frequent EMA schedules. These items are marked by * in the once-per-day schedule.

Once-per-day morning schedule

Constructs, subconstructs, and EMA items

Subconstruct	EMA item (NL)	EMA item (EN)	Response scale (NL)	Response scale (EN)	Construct	Reference
			**Main construct: Sleep**			
**Sleep**	Hoe was de kwaliteit van mijn slaap?*	What was the quality of my sleep?*	Zeer slecht – zeer goed	Not at all - very much	Sleep quality	[2]
	Ik heb gepiekerd voor ik in slaap viel*	I worried before falling asleep*	Helemaal niet - heel erg	Not at all - very much	Sleep-related worrying	Adapted from [34]
	Hoe laat ging ik naar bed?*	At what time did I go to bed?*	[selecteer tijdstip]	[select time]	Calculate sleep duration	Adapted from [34]
	Hoe laat probeerde ik in slaap te vallen?*	At what time did I try to fall asleep?*	[selecteer tijdstip]	[select time]	Calculate sleep duration	Adapted from [34]
	Hoe lang duurde het voordat ik in slaap viel?*	How long did it take before I fell asleep?*	[geef getal aan (minuten)]	[indicate number (minutes)]	Calculate sleep duration	Adapted from [34]
	Hoe vaak werd ik wakker, mijn uiteindelijke ontwaken niet meegeteld?*	How often did I wake, not counting my eventual awakening?*	[geef getal aan]	[indicate number]	Awakenings	Adapted from [34]
	Hoe lang ben ik in totaal wakker geweest?*	How long have I been awake in total?*	[geef getal aan (minuten)]	[indicate number (minutes)]	Awakenings	Adapted from [34]
	Hoe laat werd ik uiteindelijk wakker?*	At what time did I eventually wake up?*	[selecteer tijdstip]	[select time]	Calculate sleep duration	Adapted from [34]
	Hoe laat ben ik opgestaan?*	At what time did I go out of bed?*	[selecteer tijdstip]	[select time]	Calculate sleep duration	Adapted from [34]
			**Main construct: Mood**			
**Subconstruct**	**EMA item (NL)**	**EMA item (EN)**	**Response scale (NL)**	**Response scale (EN)**	**Construct**	**Reference**
**Mood**	Op dit moment voel ik me opgewekt	Right now, I feel cheerful	Helemaal niet - heel erg	Not at all - very much	PA high arousal	[2, 8-12]
	Op dit moment voel ik me somber	Right now, I feel down	Helemaal niet - heel erg	Not at all - very much	NA low arousal	[8-13]
	Op dit moment voel ik me tevreden	Right now, I feel content	Helemaal niet - heel erg	Not at all - very much	PA low arousal	[2, 8-12]

Subconstruct	EMA item (NL)	EMA item (EN)	Response scale (NL)	Response scale (EN)	Construct	Reference
	Op dit moment voel ik me geïrriteerd	Right now, I feel irritated	Helemaal niet - heel erg	Not at all - very much	NA high arousal	[2, 10-13]
	Op dit moment voel ik me energiek	Right now, I feel energetic	Helemaal niet - heel erg	Not at all - very much	PA high arousal	[8-12]
	Op dit moment voel ik me onrustig	Right now, I feel restless	Helemaal niet - heel erg	Not at all - very much	NA high arousal	[8-11]
			Main construct: Formulate your own questions			
**Subconstruct**	**EMA item (NL)**	**EMA item (EN)**	**Response scale (NL)**	**Response scale (EN)**	**Construct**	**Reference**
**Formulate your own questions**	- Op dit moment [typ hier je eigen vraag]	- Right now [enter your question here]	Helemaal niet - heel erg	Not at all - very much	Person-specific item (evening)	[21]
	- Op dit moment [typ hier je eigen vraag]	- Right now [enter your question here]	Helemaal niet - heel erg	Not at all - very much	Person-specific item (evening)	[21]
	- Op dit moment [typ hier je eigen vraag]	- Right now [enter your question here]	Helemaal niet - heel erg	Not at all - very much	Person-specific item (evening)	[21]

Note. The once-per-day schedules often are an insightful addition to the semi-random and fixed EMA schedules. Therefore, the PETRA decision aid has integrated some of the items of the once-per-day-schedules in the relevant (sub)constructs of the more frequent EMA schedules. These items are marked by * in the once-per-day schedule

References

1. Bos FM, Snippe E, Bruggeman R, Wichers M, van der Krieke L. Insights of patients and clinicians on the promise of the experience sampling method for psychiatric care. Psychiatric Services. 2019;70(11):983-91.
2. Bos FM, Snippe E, Bruggeman R, Doornbos B, Wichers M, van der Krieke L. Recommendations for the use of long-term experience sampling in bipolar disorder care: A qualitative study of patient and clinician experiences. International Journal of Bipolar Disorders. 2020;8(1):1-14. doi: https://doi.org/10.1186/s40345-020-00201-5.
3. Eisele E, Kasanova Z, Houben M. Questionnaire design and evaluation. In: Myin-Germeys I, Kuppens P, editors. The open handbook of experience sampling methodology: A step-by-step guide to designing, conducting, and analyzing ESM studies. Leuven2021.
4. Vachon H, Viechtbauer W, Rintala A, Myin-Germeys I. Compliance and Retention With the Experience Sampling Method Over the Continuum of Severe Mental Disorders: Meta-Analysis and Recommendations. JMedInternet Res. 2019;21(12):e14475.
5. Eisele G, Vachon H, Lafit G, Kuppens P, Houben M, Myin-Germeys I, et al. The effects of sampling frequency and questionnaire length on perceived burden, compliance, and careless responding in experience sampling data in a student population. Assessment. 2022;29(2):136-51.
6. Janssens KA, Bos EH, Rosmalen JG, Wichers MC, Riese H. A qualitative approach to guide choices for designing a diary study. BMC Medical Research and Methodology. 2018;18(1):1-12.
7. Palmier-Claus JE, Myin-Germeys I, Barkus E, Bentley L, Udachina A, Delespaul P, et al. Experience sampling research in individuals with mental illness: reflections and guidance. Acta PsychiatrScand. 2011;123(1):12.
8. Kramer I, Simons CJP, Hartmann JA, Menne-Lothmann C, Viechtbauer W, Peeters F, et al. A therapeutic application of the experience sampling method in the treatment of depression: a randomized controlled trial. World Psychiatry. 2014 02;13(1):68. PMID: 24497255. doi: 10.1002/wps.20090.
9. van der Krieke L, Jeronimus BF, Blaauw FJ, Wanders RBK, Emerencia AC, Schenk HM, et al. HowNutsAreTheDutch ((HoeGekIsNL): A crowdsourcing study of mental symptoms and strengths. International Journal of Methods in Psychiatric Research. 2016 06;25(2):123. PMID: 2015-44271-001. doi: 10.1002/mpr.1495.
10. Helmich MA, Snippe E, Kunkels YK, Riese H, Smit A, Wichers M. Transitions in Depression (TRANS-ID) Recovery: Study protocol for a repeated intensive longitudinal n= 1 study design to search for personalized early warning signals of critical transitions towards improvement in depression. 2021.
11. Riese H, Von Klipstein L, Schoevers R, van der Veen D, Servaas M. Personalized ESM monitoring and feedback to support psychological treatment for depression: a pragmatic randomized controlled trial (Therap-i). BMC Psychiatry. 2021;21(1):1-11.
12. Schoevers R, Van Borkulo C, Lamers F, Servaas M, Bastiaansen J, Beekman A, et al. Affect fluctuations examined with ecological momentary assessment in patients with current or remitted depression and anxiety disorders. PsycholMed. 2021;51(11):1906-15.
13. Reininghaus U, Kempton MJ, Valmaggia L, Craig TK, Garety P, Onyejiaka A, et al. Stress sensitivity, aberrant salience, and threat anticipation in early psychosis: an experience sampling study. SchizophrBull. 2016;42(3):712-22.

14. Knapen SE. Rhythm & Blues: Chronobiology in the Pathophysiology and Treatment of Mood Disorders: Rijksuniversiteit Groningen; 2019.
15. Tyler E, Jones S, Black N, Carter L-A, Barrowclough C. The relationship between bipolar disorder and cannabis use in daily life: An experience sampling study. PLoS One. 2015;10(3).
16. Myin-Germeys I, Peeters F, Havermans R, Nicolson NA, deVries MW, Delespaul P, et al. Emotional reactivity to daily life stress in psychosis and affective disorder: An experience sampling study. Acta PsychiatrScand. 2003 02;107(2):124. PMID: 2003-04279-005. PMID: 12534438. doi: 10.1034/j.1600-0447.2003.02025.x.
17. Barrantes-Vidal N, Chun CA, Myin-Germeys I, Kwapil TR. Psychometric schizotypy predicts psychotic-like, paranoid, and negative symptoms in daily life. JAbnormPsychol. 2013;122(4):1077.
18. Schreuder MJ, Groen RN, Wigman JT, Hartman CA, Wichers M. Measuring psychopathology as it unfolds in daily life: addressing key assumptions of intensive longitudinal methods in the TRAILS TRANS-ID study. BMC Psychiatry. 2020;20(1):1-14.
19. Kroeze R, van der Veen DC, Servaas MN, Bastiaansen JA, Oude Voshaar R, Borsboom D, et al. Personalized feedback on symptom dynamics of psychopathology: a proof-of-principle study. Journal for Person-Oriented Research. 2017;3(1):1-7. doi: 10.17505/jpor.2017.01.
20. Oorschot M, Kwapil T, Delespaul P, Myin-Germeys I. Momentary assessment research in psychosis. PsycholAssess. 2009 12;21(4):498. PMID: 2009-22537-005. PMID: 19947784. doi: 10.1037/a0017077.
21. Bos FM, von Klipstein L, Riese H. EMA diary items developed in PETRA pilots with clients and clinicians (2017-2021). Unpublished work. 2021.
22. Jenner JA G-JB. Groninger Lijst Omgaan met Stemmen (GLOS). Hallucinaties: Kenmerken, verklaringen, behandeling. Assen, The Netherlands: van Gorcum; 2006. p. 219-22.
23. Bos FM. Ecological Momentary Assessment as a clinical tool in psychiatry: Promise, possibilities, and pitfalls. Groningen, The Netherlands: University of Groningen; 2021.
24. Zijlema WL, Stolk RP, Löwe B, Rief W, White PD, Rosmalen JG. How to assess common somatic symptoms in large-scale studies: a systematic review of questionnaires. JPsychosomRes. 2013;74(6):459-68.
25. Rosmalen JG, van Gils A, Mesa MAA, Schoevers RA, Monden R, Hanssen DJ. Development of Grip self-help: An online patient-tailored self-help intervention for functional somatic symptoms in primary care. Internet Interventions. 2020;19:100297.
26. Wichers M, Peeters F, Rutten BPF, Jacobs N, Derom C, Thiery E, et al. A time-lagged momentary assessment study on daily life physical activity and affect. Health Psychology. 2012 03;31(2):135. PMID: 2011-22910-001. doi: 10.1037/a0025688.
27. Bringmann LF, Booij SH, Elmer T, Langener A, Stadel M, Bos FM. EMA items developed by LaBLab - in collaboration with iLab and PETRA and focus groups with clients (Unpublished work). 2021.
28. LaBLab. www.laurabringmannlab.com. [20-12-2021].
29. iLab. https://ilab-psychiatry.nl/en_US/. [20-12-2021].
30. Myin-Germeys I, Krabbendam L, Delespaul PAEG, Van Os J. Do life events have their effect on psychosis by influencing the emotional reactivity to daily life stress? PsycholMed. 2003 02;33(2):327. PMID: 2003-00513-016. PMID: 12622311. doi: 10.1017/s0033291702006785.

31. Booij SH, Wichers M, De Jonge P, Sytema S, Van Os J, Wunderink L, et al. Study protocol for a prospective cohort study examining the predictive potential of dynamic symptom networks for the onset and progression of psychosis: the Mapping Individual Routes of Risk and Resilience (Mirorr) study. BMJ Open. 2018;8(1):e019059.
32. Leverich GS, Post RM. The NIMH life chart manual for recurrent affective illness: The LCM. NIMH Monograph. 1993.
33. Schiepek G, Stöger-Schmidinger B, Kronberger H, Aichhorn W, Kratzer L, Heinz P, et al. The Therapy Process Questionnaire-Factor analysis and psychometric properties of a multidimensional self-rating scale for high-frequency monitoring of psychotherapeutic processes. Clinical psychology & psychotherapy. 2019;26(5):586-602.
34. Carney CE, Buysse DJ, Ancoli-Israel S, Edinger JD, Krystal AD, Lichstein KL, et al. The consensus sleep diary: standardizing prospective sleep self-monitoring. Sleep. 2012;35(2):287-302.
